# Incentives in prescribing, dispensing and pharmaceutical spending: A scientometric mapping.

**DOI:** 10.12688/f1000research.156306.2

**Published:** 2025-02-11

**Authors:** Tocaruncho-Ariza L. H, Riascos-Ochoa J, Jimenez-Barbosa W. G

**Affiliations:** 1 Ministerio de Salud y Protección Social de Colombia, Bogota, Colombia; 2 Universidad de Bogotá Jorge Tadeo Lozano, Faculty of Natural Sciences and Engineering, Bogotá, Bogota, Colombia; 3 Universidad de Bogotá Jorge Tadeo Lozano, Faculty of Economic and Administrative Sciences, Bogotá, Colombia

**Keywords:** Health systems, Drug financing, Price regulation, insurance, drug cost

## Abstract

**Introduction:**

Health systems worldwide are struggling to ensure the affordability of medicines. Prescription, dispensing, and pharmaceutical expenditures are key variables that highlight the need to understand how global scientific evidence is generated against factors (implicit and non-explicit) that influence these variables.

**Objectives:**

Explore and provide a detailed description of the characteristics of the global scientific production of Open Access articles related to the prescription, dispensing and pharmaceutical expenditure faced by health systems worldwide.

**Methods:**

A five-stage scientometric mapping was performed based on a systematic search of 8 databases. The five stages are: i) retrieval, ii) migration, iii) analysis, iv) visualization and v) interpretation.

**Results:**

A corpus of evidence from 103 systematic literature reviews was obtained, screened and sifted, visualizing the countries, authors, databases, journals, institutions and time periods that contributed most to evidence generation. Central research themes are identified and phenomena related to article publication are discussed.

**Conclusions:**

The analysis reveals a clear leadership of the United Kingdom and the United States in scientific production on prescribing, dispensing and pharmaceutical expenditure in health systems worldwide. This scientific production is mainly focused on financing policies, pharmaceutical incentives and interventions, and rational use of medicines. There is also evidence of the scarcity of scientific production in Latin American publications and authors, which could generate interest for future research.

## key points

Using the technique of scientometric mapping, our study characterizes the current state of global scientific production related to prescribing, dispensing, and pharmaceutical spending incentives in health systems worldwide in a rigorous and visually practical manner, providing a global and rapid overview of the dynamics of scientific literature production.

It also identifies, among other things, the main lines of publication, thematic leaders and representative authors, making it useful for other researchers or public policy makers.

## 1. Introduction

Affordability of medicines has been the focus of developing countries and global organizations, but it has now become a global issue. ^1^ In recent decades, a number of events have concerned stakeholders in different healthcare systems around the world. For example, publicly funded pharmaceutical expenditures, drug shortages, technological pressures due to new drugs, effects of the COVID-19 pandemic (shortages of raw materials or derived pathologies), and economic availability. These phenomena have created uncertainty about the financial sustainability of health systems.

Governments are developing strategies to reduce the impact of these phenomena on the efficient provision of health services. However, those strategies that involve expenditure rationalization are the most worrisome because they seem to work only for a period of time and then lose their effectiveness. This behaviour is not new, and the World Health Organization (WHO) has called for a sincere and transparent dialogue between the actors in the pharmaceutical chain in order to achieve fairer prices. ^1^

In addition to “looking for” conventional market analysis factors such as frequency and price, or those related to a holistic and etiological approach to prescribing, we delve into factors related to philosophical and anthropological concepts such as ethics, customs, power relations, professional autonomy, moral risk, ^2^ unfair competition, conflicts of interest, promotional incentives, handouts, loyalty strategies, asymmetric information, or any other factor that influences the actors in the drug chain and affects the sustainability of a health system.

These last factors become more relevant considering that a health system that obeys its coverage and efficiency in spending should mitigate the failures in achieving its objectives and strive for its sustainability and market equity. Also, the health system should evaluate the regulation of the health authority from a scenario where all stakeholders are satisfied. ^3^

To characterize define the behavior of these additional factors, it is necessary to first observe how the evidence around the world on these topics is generated. Scientometric becomes a useful tool to perform this characterization. Scientometric is a technique that visualizes the quantitative aspects of the scientific literature and it is used as a tool for the development of scientific policies of countries and organizations. ^4^

Scientometric mapping is a research method based on statistical and visualization methods to represent critical points and trends in a specific field. Therefore, it is proposed to take advantage of scientometric mappings that focus on the metrological characteristics of evidence and determine aspects such as publications, journals, countries, institutions, authors, keywords, and central research topics by defining representations of possible connections in a dynamically and continuously evolving scientific evidence system. ^5– 7^

In light of the above, the following research question arises.

What are the characteristics of the production of scientific literature on prescribing and dispensing incentives and their relationship to pharmaceutical expenditures experienced by health systems worldwide?

The results of this scientometric mapping that visualizes scientific production will be complemented in a second research with a synthesis of evidence focused on the map review to analyze the trend of scientific production identified, ^8^ and is part of a doctoral research focused on identifying and analyzing the factors that influence the cost of prescribing, dispensing and recognition costs of medicines in the Colombian health system from performance information from 2020 to 2022.

For all the above reasons, the main objective of this research is to explore and provide a detailed description of the characteristics of the world scientific production of Open Access articles related to the prescription, dispensing and pharmaceutical expenditure faced by health systems. The decision to study Open Access articles is justified by some principles of the Leiden Manifesto, ^9^ which recommends that the processes of collecting and analyzing information should be open, transparent and simple, and that the data and analysis should be open to verification by the evaluators and reviewers; in this way, any reader can access the details of all the articles included in this academic exercise and expand the information that they consider relevant.

However, it is necessary to clarify that the representativeness of tationess (OA) publications in pharmacoeconomics may be influenced through various factors, such as the countries of authorship, the impact of the journals that publish these papers, and how OA models affect the distribution and visibility of research. In general, articles related to pharmacoeconomics and the value of health interventions show a significant representation of high-income countries. ^10, 11^

Even though OA publications increase the accessibility of research, authors from low- and middle-income countries face high article processing charges (APCs), which limits their participation. ^12^ According to a study conducted in Africa and South Asia, APCs can reach up to ten times the magnitude of those in developed countries. ^13^ Despite these challenges, many journals now offer discounted rates for authors from low- and middle-income countries. ^14^

Open access journals play a key role in global dissemination, as they reach a wider audience through online access. This translates into increased citation rates, ^15^ however, this model has challenges such as high costs and uneven quality. ^16, 17^ Therefore, it is clear that while open access publications increase the visibility and citation of research, significant challenges remain that affect the equity and representation of authors from middle and low income countries, so the results should be treated with caution.

## 2. Methods

### 2.1 Study design

This research has a exploratory scope developed by scientometric mapping, which includes five main stages ^4, 18^: recovery or selection of sources, migration or extraction of data, analysis, visualization and interpretation.

### 2.2 Pre-recovery stage

A structured and rigorous research protocol was proposed, decomposing the research question into each of its components using the PICO structure (population, intervention, comparison and outcomes) ^19^:

Population: The study should include at least one of the following knowledge users:

2.2.a Government decision makers

2.2.b Policy advisors responsible for data analysis and reporting and recommendations for decision making

2.2.c Administrators or stakeholders in public or private health systems, both at the level of policy implementers and providers

2.2.d Scientific groups or societies

2.2.e Users or patients

Intervention: Studies will be considered that describe any strategy related to the analysis of prescription, dispensing, incentives and pharmaceutical expenditure of medicines in a health care setting.

Comparison: Articles with or without a comparison group were eligible for inclusion.

Outcome: The selected outcomes of interest were those related to the identification of strategies, facilitators, barriers, and any other factors that allow establishing the relationship between prescribing, dispensing, incentives, and pharmaceutical expenditures.

### 2.3 Eligibility criteria

2.3.1 Inclusion criteria

2.3.1.a Criteria for inclusion of studies in terms of format, language, and date of publication.

- Article available as final publication, open access.
- No language restriction.
- No publication date restriction.

2.3.1.b Criteria for study inclusion regarding design, methodological and reporting quality.

- Type of study: Systematic Literature Reviews (SLR), which provide reliable information for health decision-making due to their rigorous and reproducible methodology, with specific objectives and eligibility criteria for the evidence, a systematic search and subsequent assessment of the validity of the results, and a structured synthesis. Compared with primary studies, SLR reduces the risk of bias in the selection and analysis of evidence, has less random error and greater statistical power when combining the results of different primary studies under comparison. ^20^

2.3.2 Exclusion criteria

Letters to the editor, editorials, posters and comments were not included.

## 3. Recovery

This phase included the following activities:

### 3.1 Selection of information sources

Databases were selected based on their bibliographic content and relevance to the research topic: Cochrane Database of Systematic Reviews (Wiley platform) with systematic reviews of controlled clinical trials and other studies, ^21^ MEDLINE (Ovid platform or PubMed) for its amount of biomedical bibliography and to be of greater use worldwide, ^22^ TRIP DATABASE (Turning Research Into Practice) ( https://www.tripdatabase.com/, s.f.) specialized in clinical research, Science direct (Elsevier platform) ^23^ with a multidisciplinary and peer-reviewed approach, EPISTEMONIKOS cataloged as a health evidence search engine with a large number of SLRs, ^24^ the University of York: Database of Center Reviews Dissemination, which groups 3 databases that include SLR with previous quality review, ^25^ some Latin American databases such as LILACS (Virtual Health Library - VHL) or international open access collaborations such as SCIELO (Scientific Electronic Library Online), and finally Google Scholar for complementary and optional evidence if needed.

The literature search was conducted on July 19, 2023 according to the search strategies described below.

### 3.2 Design and implementation of electronic search strategies

Natural language key words were identified from the research question. Search strategies were designed using DeCS controlled vocabulary (MeSH) and uncontrolled vocabulary (free language). The strategies were supplemented with the field identifiers title and abstract (.tw.), Boolean operators (OR and AND) and/or methodological filters according to the requirements of each database, without restriction of publication date or language, and full text available in open access.

### 3.3 Study selection process

The search strategies and results were stored in electronic format (Appendix 1. Search strategies) using a matrix previously designed in the Microsoft Excel ^®^ program. The first stage of article selection was performed by screening by reading the title and abstract and eliminating duplicates (Appendix 2. References deleted for duplicity).

### 3.4 Description of studies, screening of references and selection of studies

Full text screening was performed using the eligibility criteria. The results of the reference screening and study selection are presented in a process flow diagram (Appendix 3). In addition, the list of excluded studies and the reason for exclusion (Appendix 4) and the studies included in the research corpus (Appendix 5) are attached.

## 4. Migration. Data extraction and synthesis of evidence

In a standard format designed a priori in the Microsoft Excel ^®^ program, the main data of each selected article and the findings of interest in relation to the outcomes proposed for this research were included, extracted by an expert reviewer in the field (Appendix 6. Data extraction and evidence synthesis variables).

## 5. Results

426 references were screened and 75 of them were excluded for duplicity. The remaining were read in title and full text as evidence screening, applying the eligibility criteria and excluding 249 studies for not meeting them. Finally, 103 articles to be included in the research are obtained.

### 5.1 Analysis

Since scientometric mapping is the quantitative processing of evidence, ^26^ with the corpus of 103 selected references (hereinafter, research corpus), and from the 40 variables of the previously defined matrix (Appendix 6. Data extraction variables and evidence synthesis) were processed using electronic Microsoft Excel sheets, different relational tables of the variables (e.g., journal and country of origin of the publication, date of publication, affiliation of the lead author, funder), population characteristics and contextual factors (e.g., country of application, type of drug analyzed), and general findings of the SLR as a central theme, crossing this information with other sources to continue with the evidence visualization phase.

## 6. Visualization

The results were presented considering the topics that could answer the research question and the general objective. For visualisation, all graphs presented in this section were created using Microsoft Excel 2019 MSO (16.0.10398.20008) 64-bit and the following free online software:

a. Tableau Public. Available at: https://www.tableau.com/products/public
b. Power BI (Business Intelligence). Available at: https://www.microsoft.com/es-es/power-platform/products/power-bi

c. Gephi. Available at https://gephi.org/
d. Rawgraphs 2.0. Available at: https://www.rawgraphs.io/
e. Word cloud from: https://www.nubedepalabras.es/
f. Word cloud from: https://voyant-tools.org/

The themes analyzed were

a. Databases and publications
b. Authors
c. Citations
d. Journals and Publications
e. Institutions and Countries
f. Funders and Sponsors
g. Co-occurrence of terms
h. Research trends

### 6.1 Databases and publications

**Figure 1.**
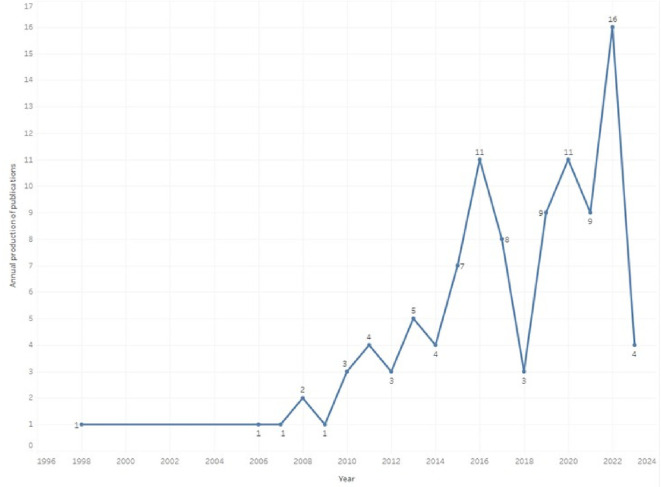
The annual production of publications.

Scientific production related to prescription, dispensing and pharmaceutical expenditure is found from 1998 with the publication of an article ( Figure 1), then a period in which publications were not identified until 2006, where again evidence was found related, although in the same number of annual generation, with an annual growth trend since 2010, reaching its highest value in 2022. The 2023 values are presented only up to the date of the evidence search carried out in July 2023, so this year’s data do not correspond to the entire annuity, but it was decided to include them because, contrary to the observed increasing trend, in seven months of 2023 only four articles were found. As of 2018, more than half of the total scientific output is concentrated in the research topic (52%).

Compared to the number of primary studies included in the research corpus, according to the data from Table 1, it was observed that the SLRs generally develop a broad search for evidence with an initial total harvest of more than 720,000 studies or primary sources, but when applying the screening and selection process, less than 1% of the total evidence recovered in each SLR is selected. These proportions are similar across all SLRs.

**Table 1.** Relationship between primary studies from initial searches and included studies after screening.

Year of publication	Number of publications	Total primary studies recovered in initial search	Total studies included in RSL	Inclusion ratio
1998	1	No report	43	-
2006	1	2.009	6	0.30%
2007	1	1.903	15	0.79%
2008	2	No report	204	-
2009	1	2.036	28	1.38%
2010	3	3.246	54	1.66%
2011	4	10.622	63	0.59%
2012	3	6.475	51	0.79%
2013	5	13.979	161	1.15%
2014	4	40.890	105	0.26%
2015	7	37.372	404	1.08%
2016	11	81.402	168	0.21%
2017	8	29.723	214	0.72%
2018	3	3.589	47	1.31%
2019	9	57.561	502	0.87%
2020	11	64.687	358	0.55%
2021	9	162.208	425	0.26%
2022	16	136.480	765	0.56%
2023	4	65.943	620	0.94%
**TOTAL**	**103**	**720.125**	**4.233**	**0.59%**

**Figure 2.**
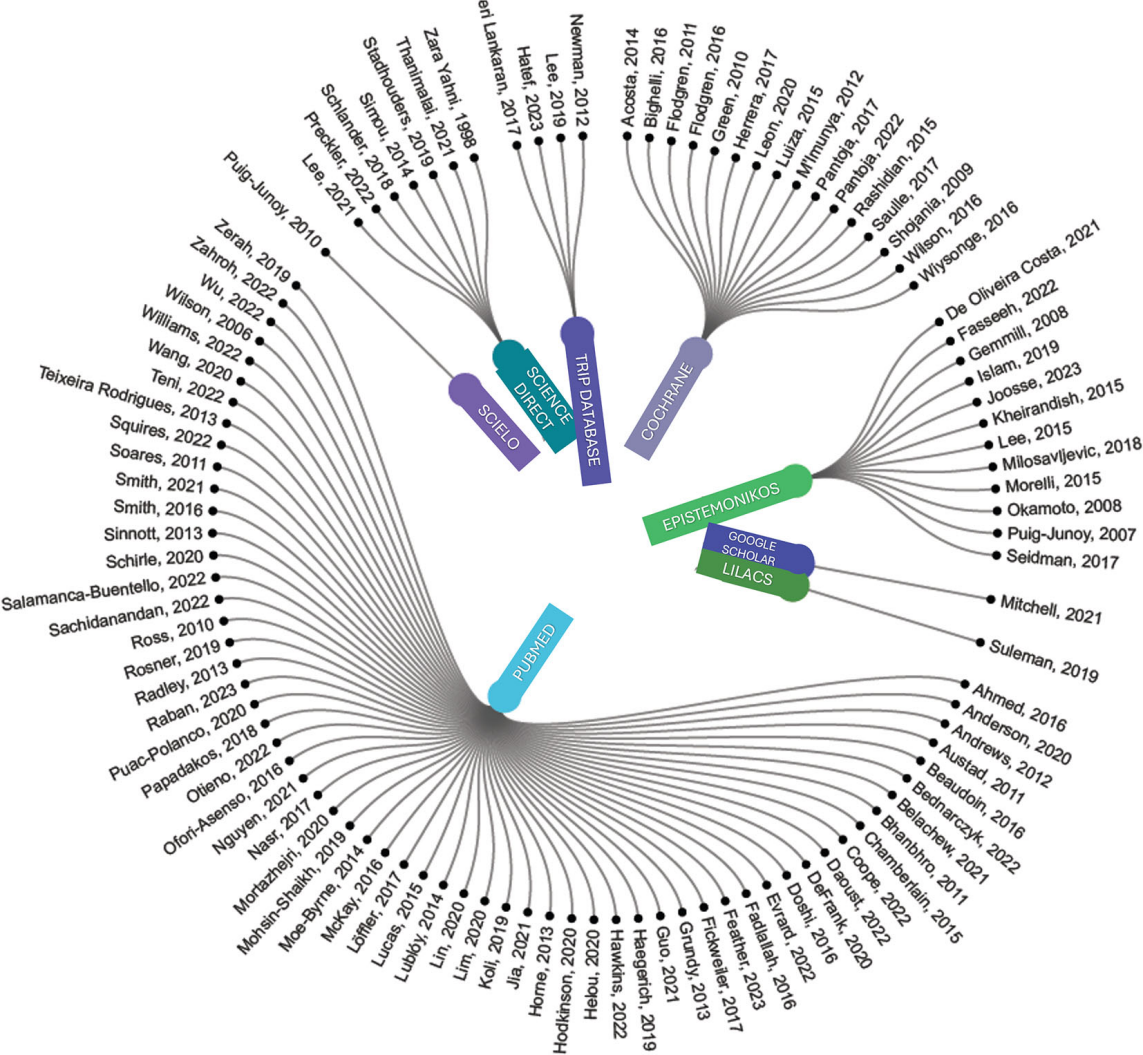
Circular dendrogram on scientific output by year and database, in the research theme.

The annual scientific output per database consulted is summarized in Figure 2, where PUBMED presents the highest proportion of evidence with 61 articles (59.22%) of the research corpus covering the longest period of published articles (2006-2023). It is followed by Cochrane with 16 publications (15.53%) from 2009 to 2022 and Epistemonikos with 12 articles (11.65%) from 2007 to 2023.

### 6.2 Authors

All authors (main or co-authors) reported in the research corpus were listed, resulting in a total of 517 authors. The productivity of authors was analyzed using Lotka’s law, first proposed in 1926, ^27^ which refers to a quantitative relationship between authors and contributions produced in a field over a given period of time. The interpretation of this productivity index given by Del Valle Salinas ^28^ is as follows:

Productivity level (PN): Low 0, Result: 0

Productivity level (PN): Medium >0<1, Result: >0<1

Productivity level (PN): High >1, Result: >1

The application of the calculation of this index was obtained as Salinas, ^28^ and is shown in Table 2:

**Table 2.** Lotka productivity index for authors.

(n) Number of items	Number of authors	% Number of authors	Apparent items	Lotka Productivity Index (lg10n)
1	486	94.00%	486	-
2	22	4.26%	44	0.301
3	8	1.55%	24	0.4771
5	1	0.19%	5	0.699
	**517**		**559**	

Source: Authors' own elaboration from Del Valle Salinas (2018) and data from the research corpus.

It was obtained for the 517 authors appearing in the research corpus:

Low productivity 486 (94.00%) Authors with PN: 0

Medium productivity 31 (6.00%) authors with 0<PN<1

High productivity 0 (0.00%) Authors with PN>1

**Figure 3.**
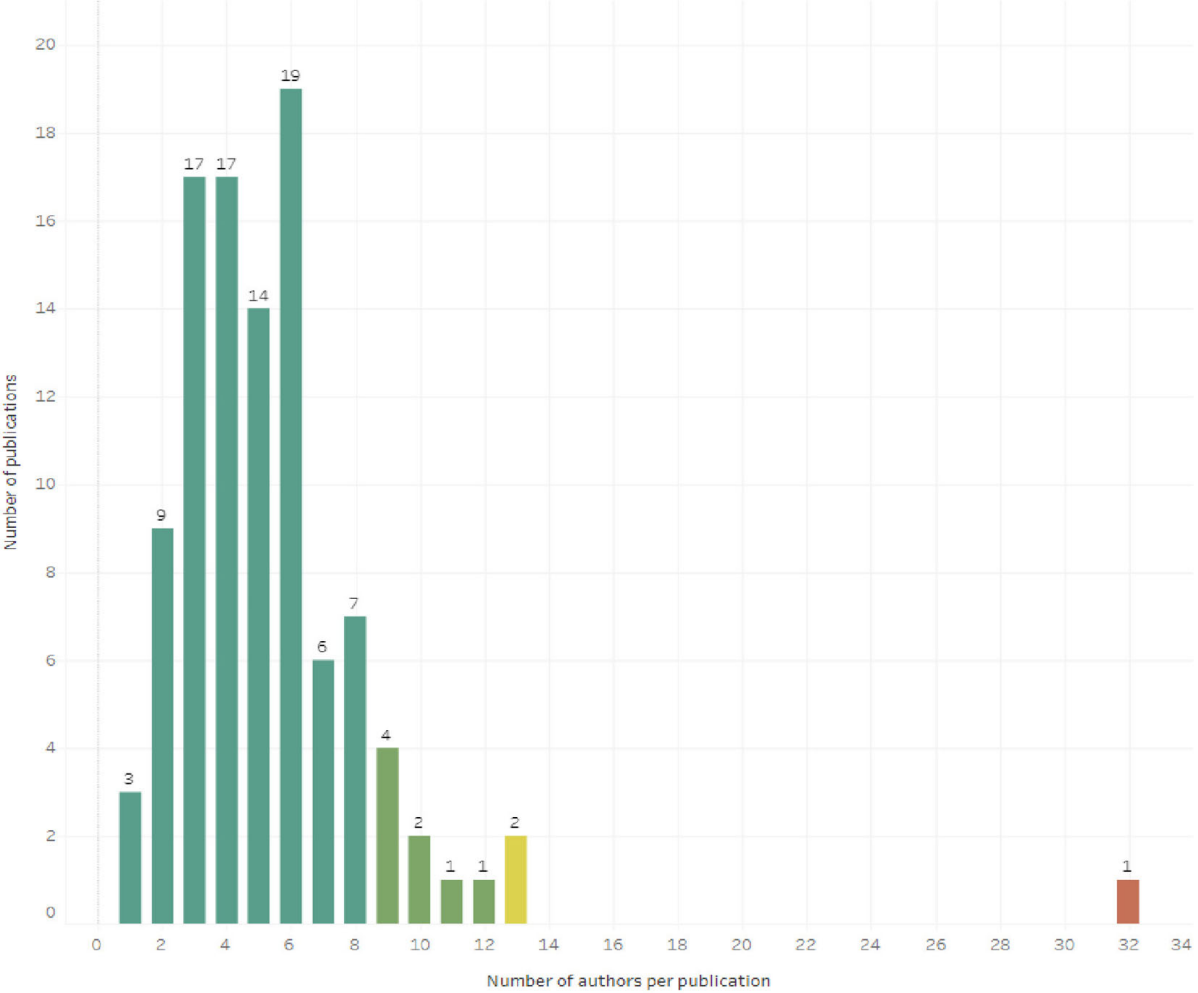
Histogram for the number of authors per article.

As for the collaboration between authors, only 2.91% of the articles report a single author (3 articles), while the concentration of authors between 3 and 6 authors is concentrated in 67.00% of the 103 articles. One of the 103 articles with 32 authors is a SLR conducted in Canada in 2022 (lead author: Squires JE) on the inappropriate use of clinical practices, overuse and underuse of medications, and includes a section with detailed contributions by name of 10 of the 32 authors ( Figure 3).

**Figure 4.**
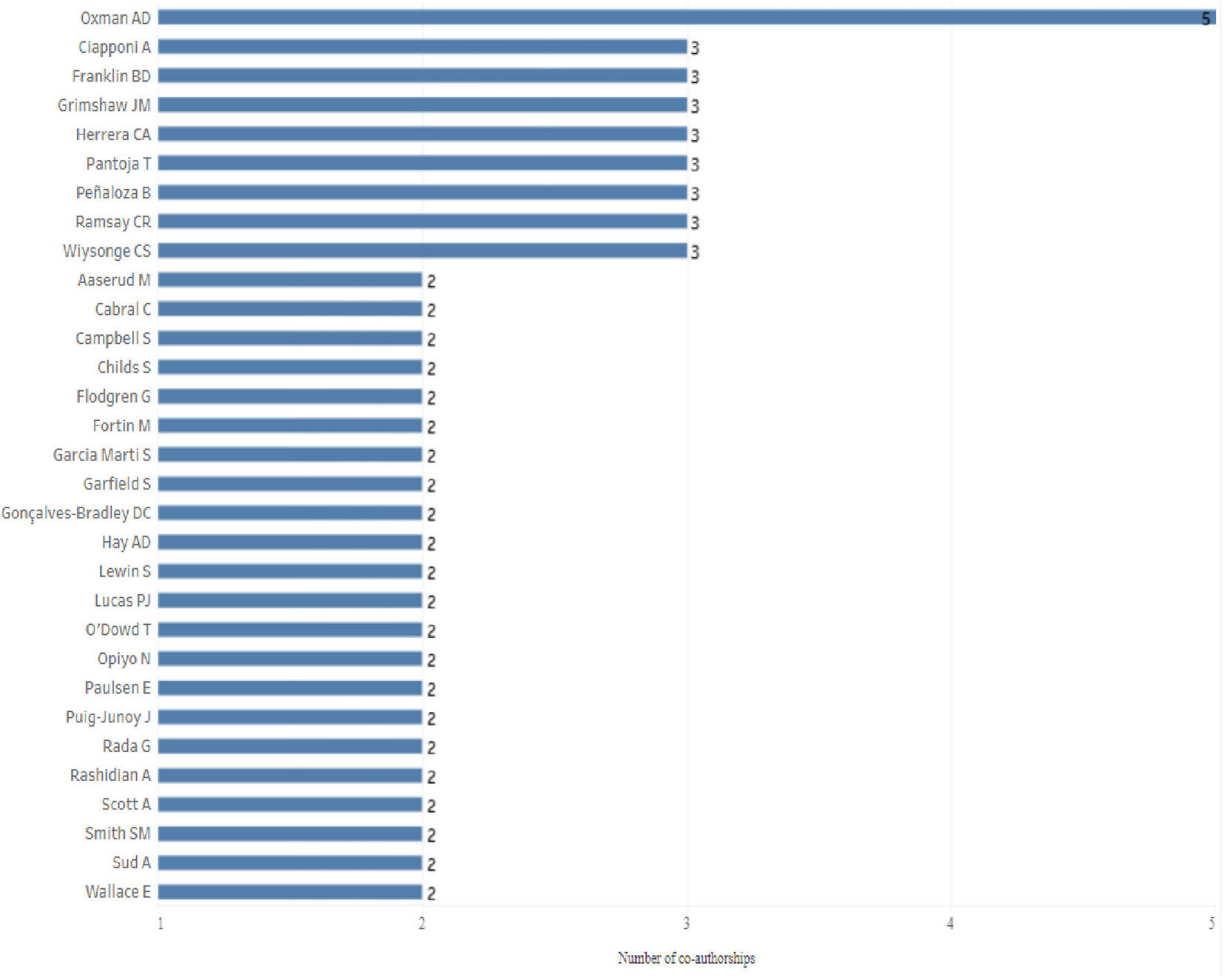
Bar chart for authors with 2 or more co-authorships in the study period 1998-2023.

Only one of the 517 authors (Oxman A.D.) appears in 5 articles of the research corpus, in all of them in the last place of the signature of the authors, which denotes his role as director of each of the SLR according to the interpretation FLAE: “first-last-author-emphasis”, ^29^ all of them “Cochrane reviews” published in the Cochrane Database of Systematic Review. 8 authors sign in 3 articles, of which 1 of them (Franklin BD) presents the same situation as Oxman AD when he is the last author in the three articles, that is, as director, while in the remaining 7 cases the order in the signatures of authorship is presented as main author or co-author. Special cases are Grimshaw JM, Peñaloza B, Pantoja T and Herrera CA, as they are all present in the same 3 articles ( Figure 4).

22 authors sign co-authorships in 2 different articles of the research corpus, and the remaining 486 authors appear in only one article, that is, only 5.99% of the total authors are in the signature of authors in 2 or more articles.

**Figure 5.**
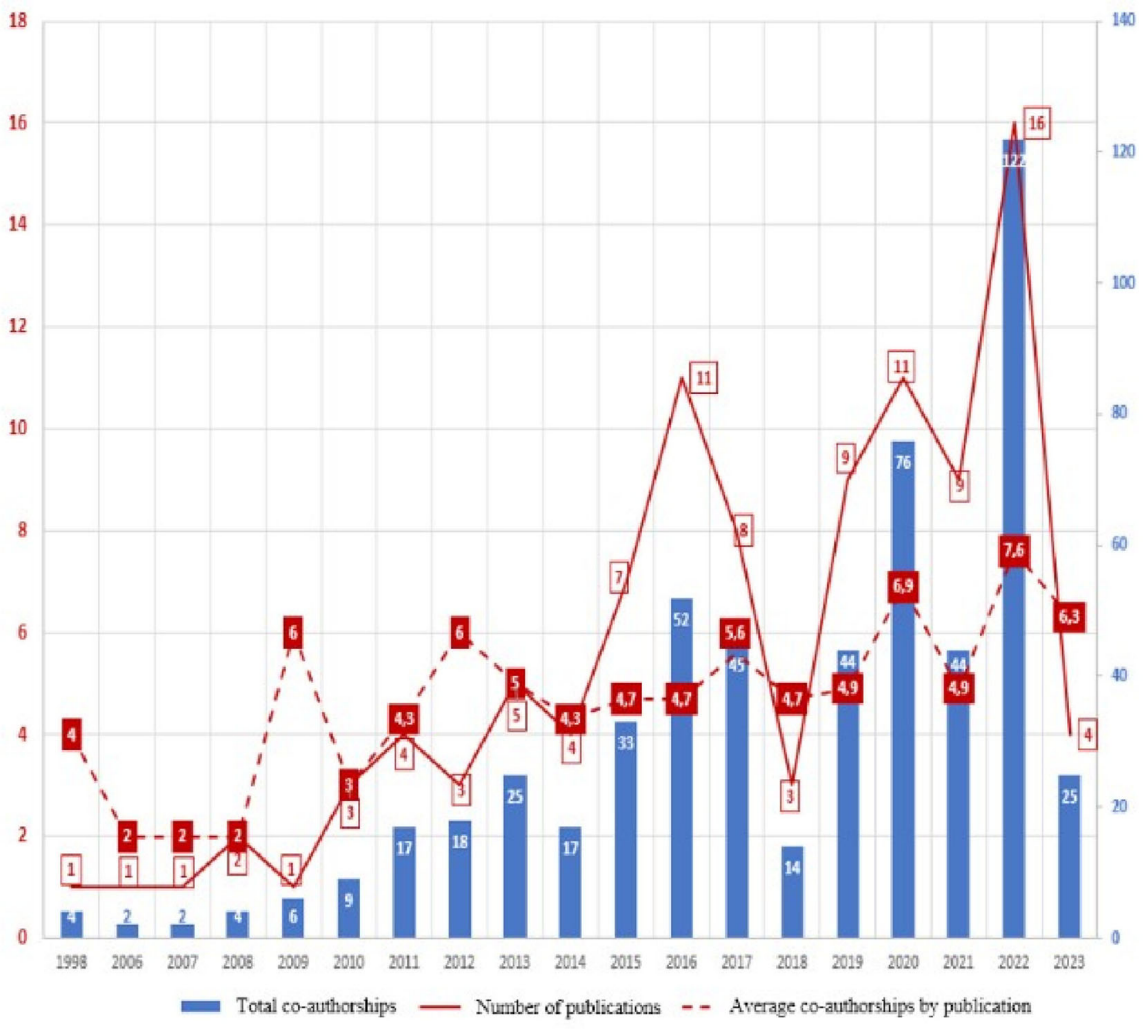
Comparative histogram of annual co-authorships and average per article vs. annual publication production.

By visualizing the total number of authors/co-authors per year and calculating the average number of authors/co-authors, we obtain the behavior of Figure 5, where we find that the number of authors/co-authors is directly proportional to the number of articles published in each year, but the average number of authors/co-authors is more stable, ranging between 4 and 6 authors per article ( Figure 5).

### 6.3 Citations

In identifying which publications have greater visibility in the global evidence, a limitation was found since the databases used do not allow in all cases to know the number of citations, therefore they were quantified from the search in the same data source in which all the selected articles were found, using the citation that appears in Google Scholar, and recognizing that some authors have criticized the lack of rigor in the number of citations presented because it is not always accurate, either because there is no significant degree of duplication, because of the opacity in the selection of sources or because it could be susceptible to manipulation. ^30^

**Figure 6.**
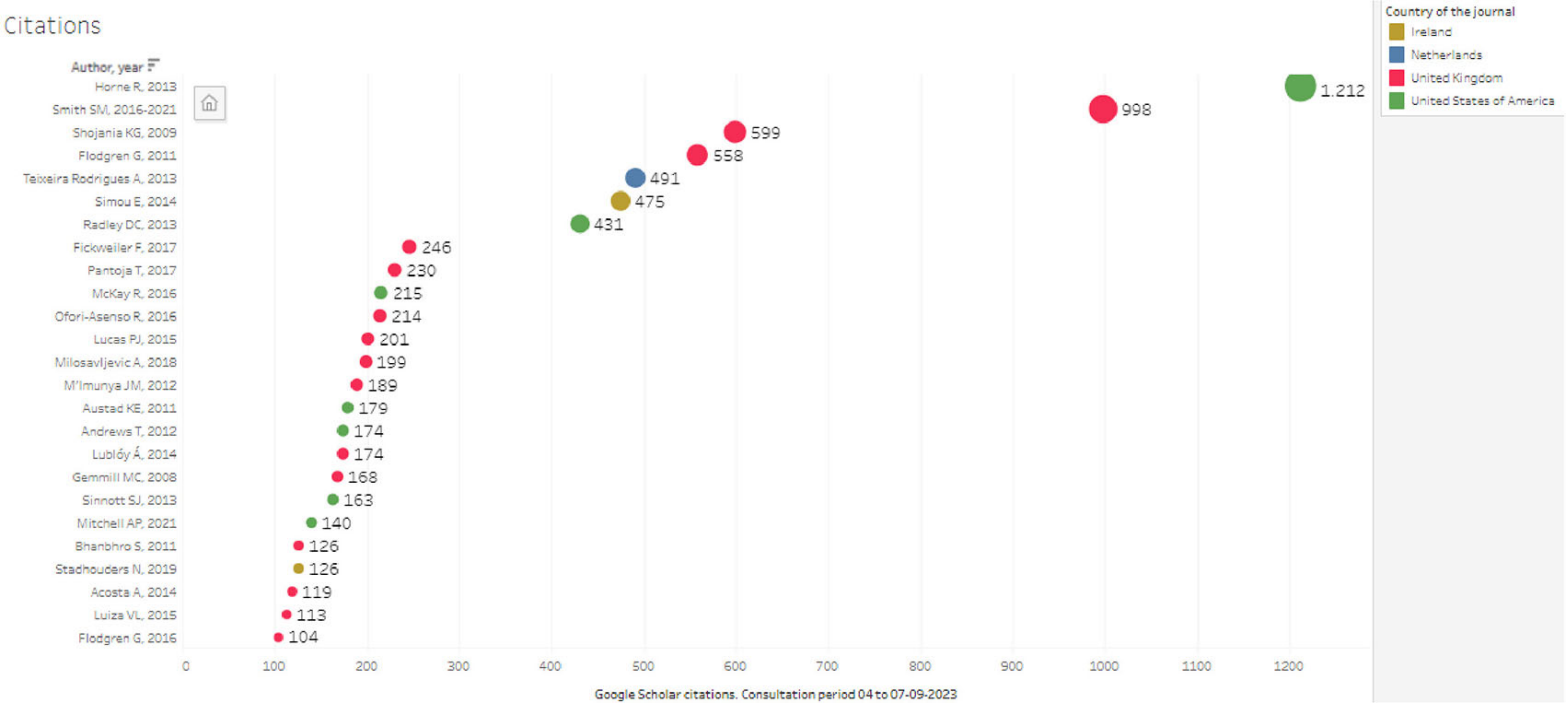
Top articles representing 80% of the total citations of the 103 SLR.

When looking at articles by number of citations and country of publication, the largest number of citations was concentrated in the last quartile of citations greater than 104, which coincides with articles representing 80% of the total citations, standing out in the top of Figure 6, Horne R.’s article with 1,212 citations, followed by Smith S.M., with 998 citations (with 2 publications, in 2016 and an update in 2021, which was not defined as a duplicate in the screening but is considered as a single publication for this analysis), and Shojania KG, with 5. 99; then Flodgren G, Teixeira Rodrigues, Simou and Radley, all with citations greater than 400, thus limiting the countries of the journals of publication of 13 in the research corpus to only 4 of the top 10, where it is the United Kingdom that together represents more than 2600 citations in five publications, followed by the United States with slightly more than 1800 citations in three articles. Table 3 shows the details of the publications with the most citations and variables and the countries where the primary studies were developed.

**Table 3.** Top 10 most cited publications in Journals.

Top	Title	Authors	Year of publication	Citations	Name of the Journal	Country of the Journal	Countries of application of primary SLR studies
				Google Scholar			
				Search:			
				04-07/09/2023			
1	Understanding patients' adherence-related beliefs about medicines prescribed for long-term conditions: a meta-analytic review of the Necessity-Concerns Framework.	Horne R, Chapman Sc, Parham R, Freemantle N, Forbes A, Cooper V.	2013	1212	PLoS One	United States of America	17: Australia, Denmark, Egypt, Estonia, France, Germany, Ireland, Italy, Japan, Netherlands, New Zealand, Singapore, Spain, Sweden, Swaziland, United Kingdom, United States of America.
2	Interventions for improving outcomes in patients with multimorbidity in primary care and community settings.	Smith Sm, Wallace E, O'Dowd T, Fortin M.	2016-2021	998	Cochrane Database of Systematic Reviews	United Kingdom	5: Australia, Canada, Netherlands, United Kingdom, United States of America.
3	Interventions for improving outcomes in patients with multimorbidity in primary care and community settings. [Update]	Smith Sm, Wallace E, O'Dowd T, Fortin M.	2021	998	Cochrane Database of Systematic Reviews	United Kingdom	3: Australia, United Kingdom, United States of America.
4	The effects of on-screen, point of care computer reminders on processes and outcomes of care	Kaveh G Shojania, Alison Jennings, Alain Mayhew, Craig R Ramsay, Martin P Eccles, Jeremy Grimshaw	2009	599	Cochrane Database of Systematic Reviews	United Kingdom	23: Australia, Bulgaria, Canada, China, Croatia, Denmark, Israel, Italy, Germany, Latvia, Montenegro, Netherlands, New Zealand, Norway, Poland, Portugal, Romania, Serbia, Spain, Sweden, Switzerland, United Kingdom, United States of America.
5	An overview of reviews evaluating the effectiveness of financial incentives in changing healthcare professional behaviours and patient outcomes (Review)	Flodgren G, Eccles Mp, Shepperd S, Scott A, Parmelli E, Beyer Fr	2011	558	Cochrane Database of Systematic Reviews	United Kingdom	2: United Kingdom, United States of America.
6	Understanding physician antibiotic prescribing behaviour: a systematic review of qualitative studies.	Teixeira Rodrigues A, Roque F, Falcao A, Figueiras A, Herdeiro Mt.	2013	491	International Journal of Antimicrobial Agents	Netherlands	12: Belgium, Canada, China, Peru, Germany, India, Iceland, Italy, Spain, Switzerland, Thailand, United States of America.
7	Effects of the economic crisis on health and healthcare in Greece in the literature from 2009 to 2013: a systematic review.	Simou E, Koutsogeorgou E.	2014	475	Health Policy	Ireland	4: Austria, Greece, Ireland, United Kingdom.
8	Reduction in medication errors in hospitals due to adoption of computerized provider order entry systems.	Radley Dc, Wasserman Mr, Olsho Le, Shoemaker Sj, Spranca Md, Bradshaw B.	2013	431	Journal of the American Medical Informatics Association: JAMIA	United States of America	1: United States of America
9	Interactions between physicians and the pharmaceutical industry generally and sales representatives specifically and their association with physicians' attitudes and prescribing habits: a systematic review.	Fickweiler F, Fickweiler W, Urbach E.	2017	246	BMJ Open	United Kingdom	18: Bangladesh, Brazil, Canada, Egypt, Ethiopia, France, Germany, India, Iran, Libya, Japan, New Zealand, Pakistan, Peru, Poland, Slovenia, Poland, Saudi Arabia, United States of America.
10	Implementation strategies for health systems in low-income countries: an overview of systematic reviews (Review)	Pantoja T, Opiyo N, Lewin S, Paulsen E, Ciapponi A, Wiysonge Cs, Herrera Ca, Rada G, Peñaloza	2017	230	Cochrane Database of Systematic Reviews	United Kingdom	(5) Australia, Canada, Netherlands, United Kingdom, United States of America.
		B, Dudley L, Gagnon Mp, Garcia Marti S, Oxman Ad					

Source: Authors' own elaboration from the research corpus and Scimago Journal & Country Rank 2022 (Scimago Journal & Country Rank., n. d.).

### 6.4 Journals and publications

In order to find out how the research corpus behaves according to the journals in which it is published, Bradford’s law was used first, as follows:

6.4.1 Bradford’s Law

First proposed in 1934 and published in 1948, this law states that the production of articles in journals presents an uneven distribution, where a high number of articles are concentrated in a small number of journals and, conversely, a small number of articles are distributed in a large number of journals. ^31, 32^ Its graphical representation is made by 3 areas, each representing 33% of the publications, and estimates the exponential decrease in performance when expanding the bibliographic search in publishing journals.

For the research corpus, the core and each domain were defined with 34,33 articles, resulting in a descending order of publications in each journal, as shown in Table 4:

**Table 4.** Bradford law enforcement results.

Bradford zonal distribution	Number of publications	Number of publishing journals
Core	35	3
Zone 2	34	22
Zone 3	34	34
**Total**	**103**	**59**

Source: Authors' own elaboration.

**Figure 7.**
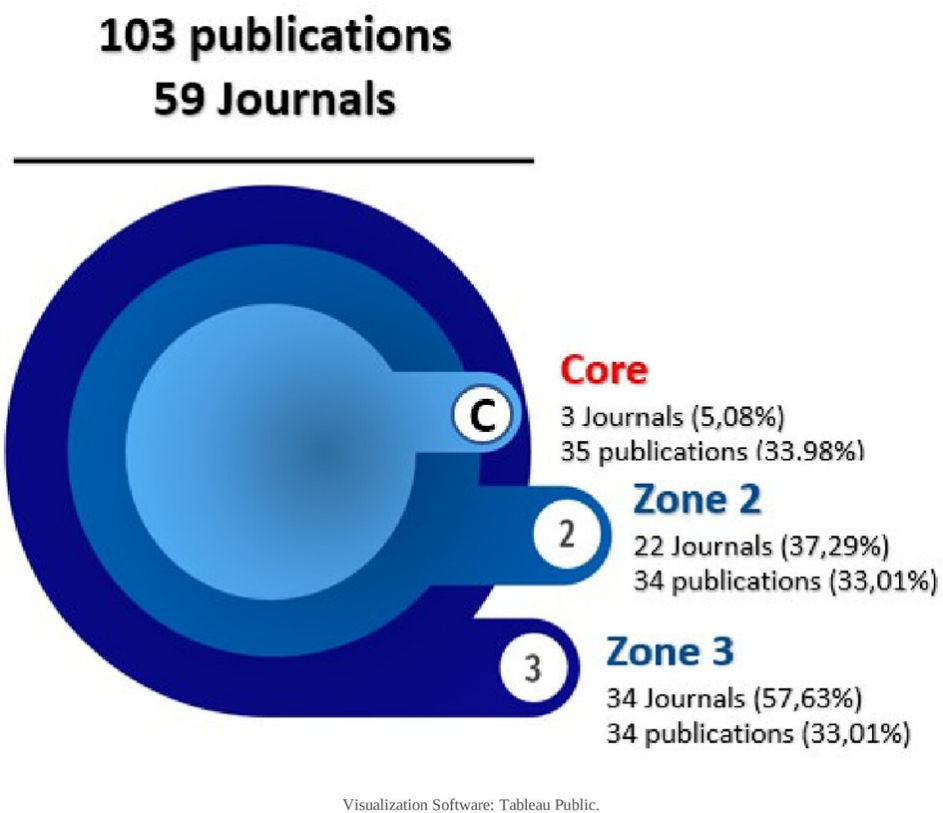
Bradford ring scatter plot of scientific production related to prescription, dispensing and pharmaceutical expenditure.

**Figure 8.**
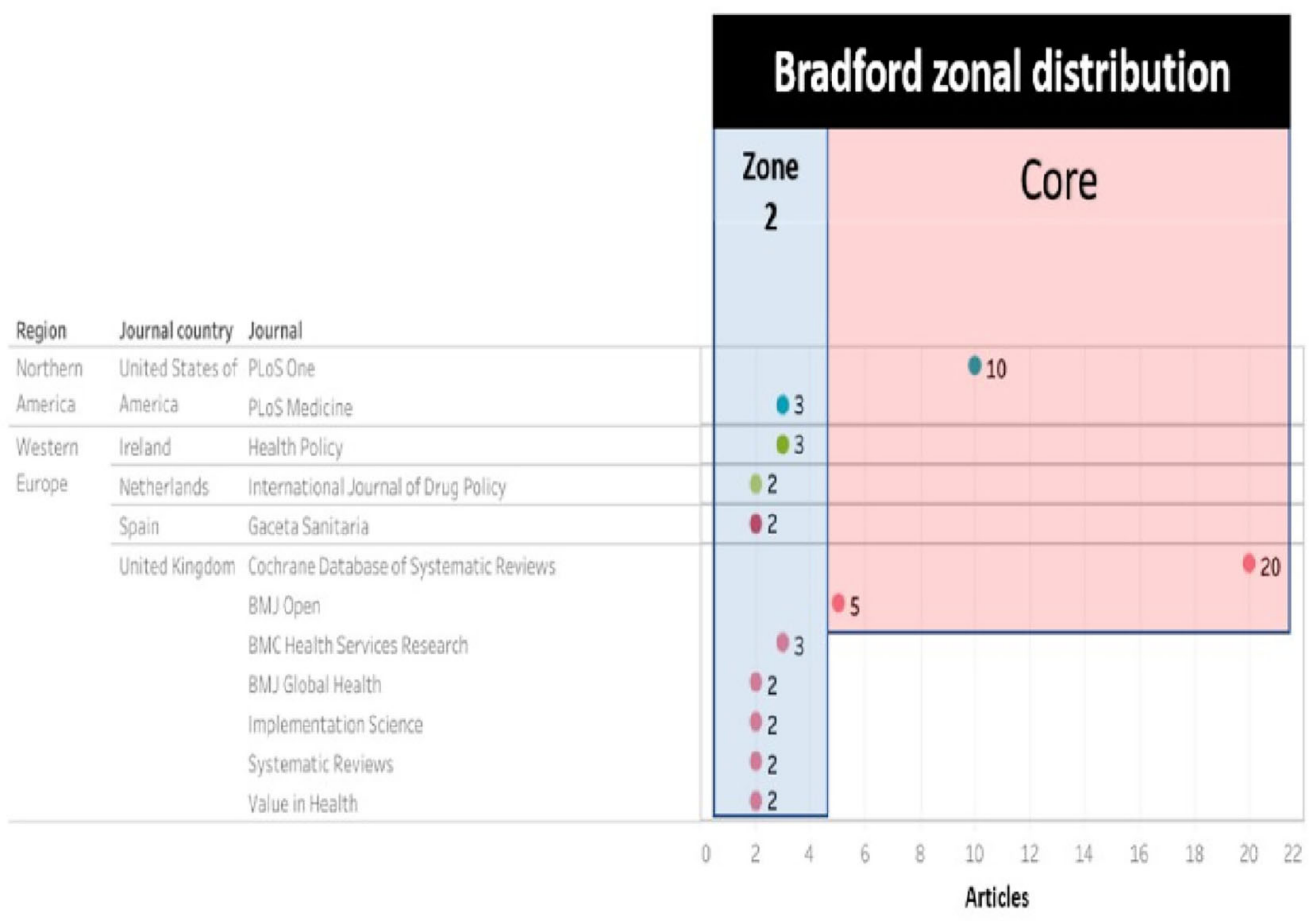
Journals with 2 or more publications.

And with these data visualized as shown in Figures 7 and 8, only 3 journals concentrate 33.98% of the SLR, then in increasing order, 22 journals and then 34 journals concentrate the next 33% of the SLR. The core is: Cochrane Database of Systematic Reviews (CDSR), PLoS One and BMJ Open as publishing journals.

**Figure 9.**
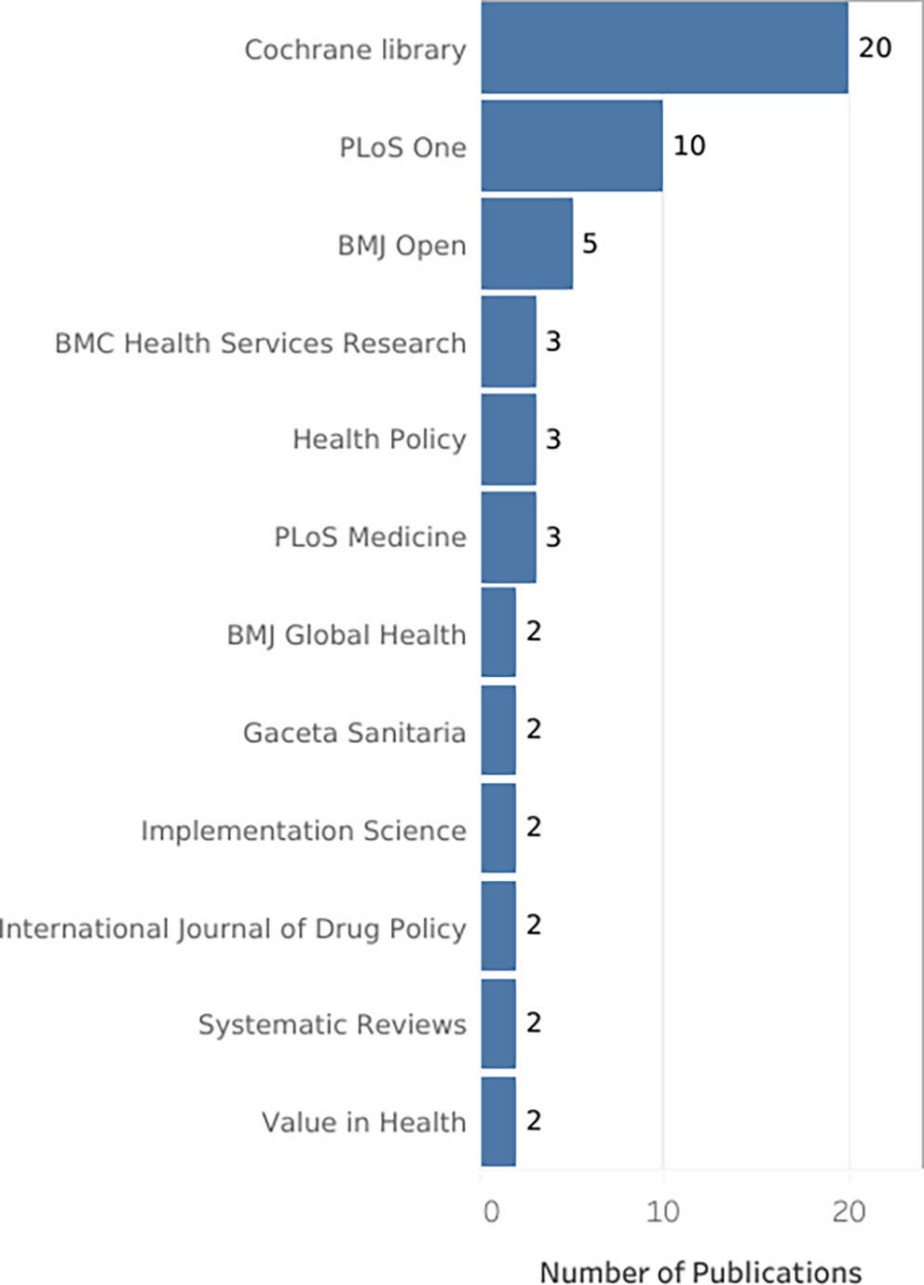
Bar chart. Journals with the highest number of retrieved publications by research topic.

**Figure 10.**
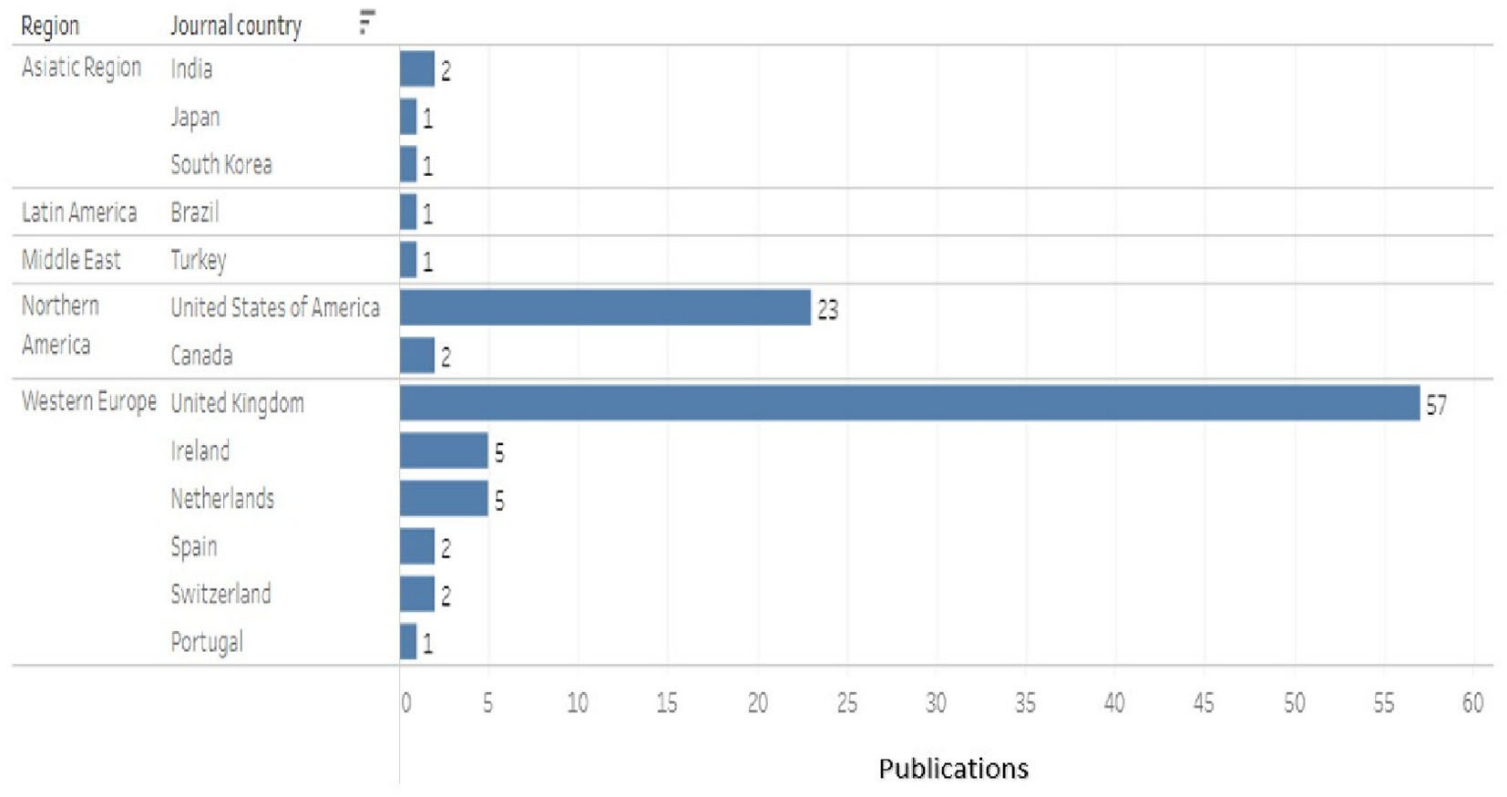
Bar chart of publications by region and country of journal (D).

The top journals (with 2 or more articles) were visualized in Figure 9, defining which belong to the core (Cochrane Database of Systematic Reviews, Plos One and BMJ Open) and which belong to zone 2 according to Bradford’s law. Figure 10 ranks the top among the total number of journals in which the research corpus was published, with a high publication difference compared to other journals:.

Second, the results obtained by region and country with more publications according to the journal are shown in Figure 10, and being CDSR and BMJ Open journals from the United Kingdom, the highest proportion of publications, more than 50% of the total, was observed in the region Western Europe and the country United Kingdom.

The regions and countries of each journal correspond to the ranking in Scimago Journal & Country Rank 2022. ^33^

6.4.2 Impact factor

The impact factor of each journal was visualized, according to the assigned quartile in Scimago Journal & Country Rank 2022, as an evaluation that the scientific community has about the journals in which the research corpus was published, which gives indications of the quality and relevance that has for an author to publish in them, based on their relative position in their field of study and, for this purpose, the assigned impact factor in the classification that Scimago Journal & Country Rank 2022 was used.

**Figure 11.**
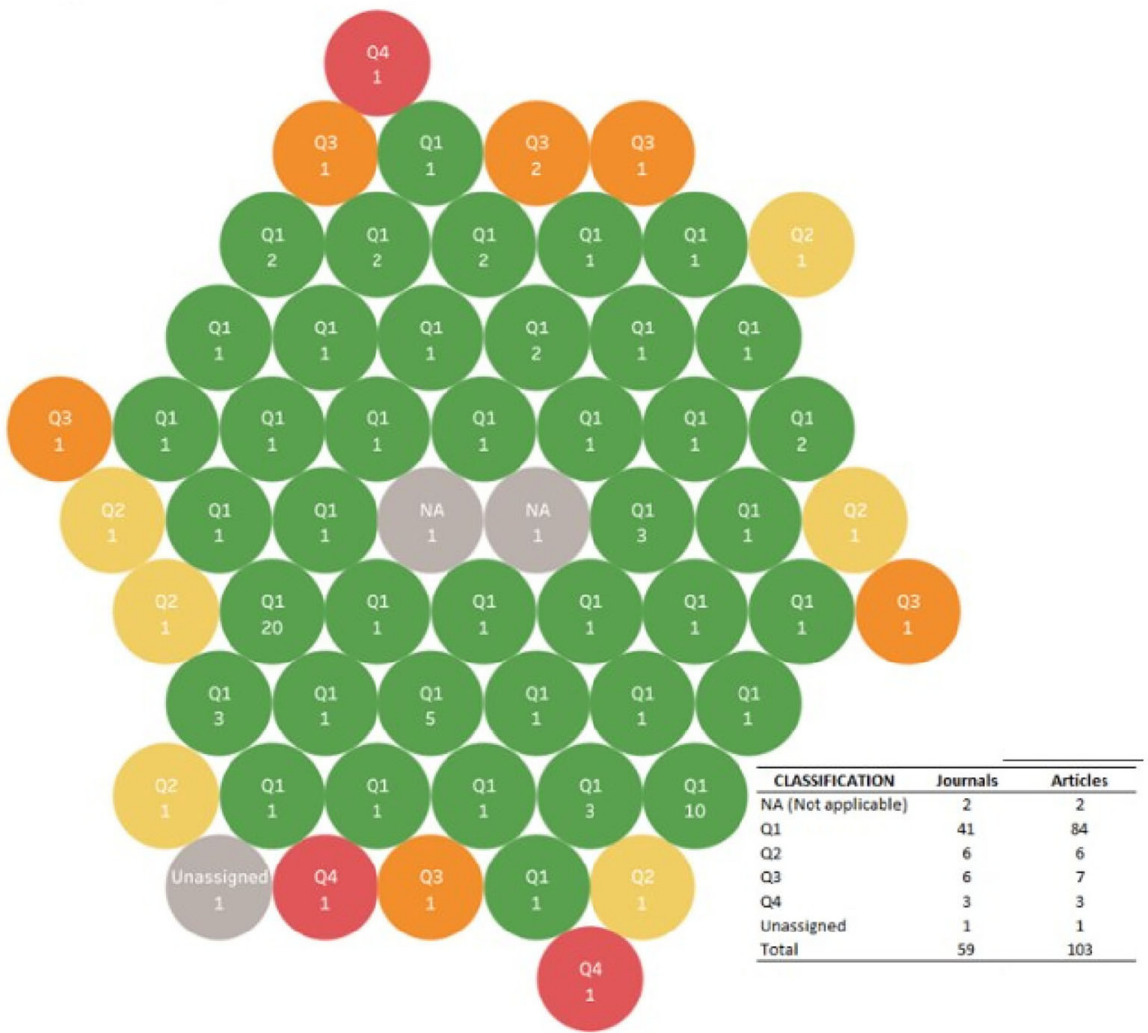
Bubble chart for quartile of journal of publication, and the number of related articles.

In this way, as shown in Figure 11, of the 59 journals in which the research corpus was published, approximately 70% of them were classified in the first quartile, that is, they have the highest impact index, followed by 10% in the second quartile.

Only in 3 cases of the reviews included in the corpus, the assigned quartile was not found, because it was not a publication in an indexed journal, but in a database: Newman M, published in 2012, and Hatef E, published in 2022, or because it had not been assigned at the time of analysis, as was the case with the publication of Okamoto E in 2007.

6.4.3 Country of origin of the publication journal

**Figure 12.**
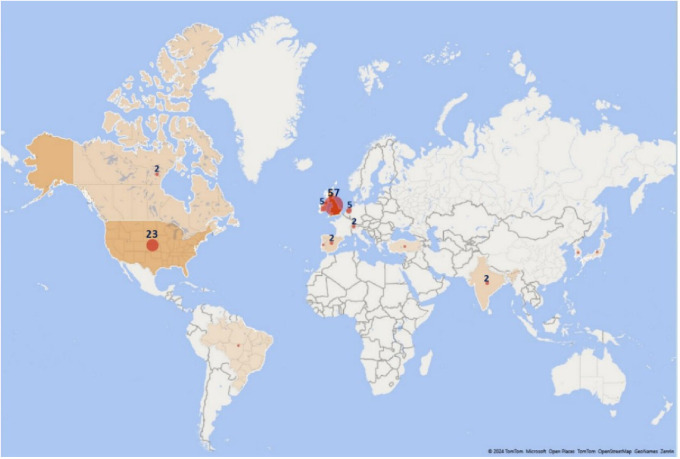
Publications by journal origin.

The geographical origin of each journal was used, finding that the results maintain the same trend of previous results, leading the United Kingdom with more than 50% of the total publications, followed by the United States of America with 23%. The trend is higher in Europe than in the Americas, where the United States dominates. Only one article published in a South American journal was recovered ( Figure 12).

**Figure 13.**
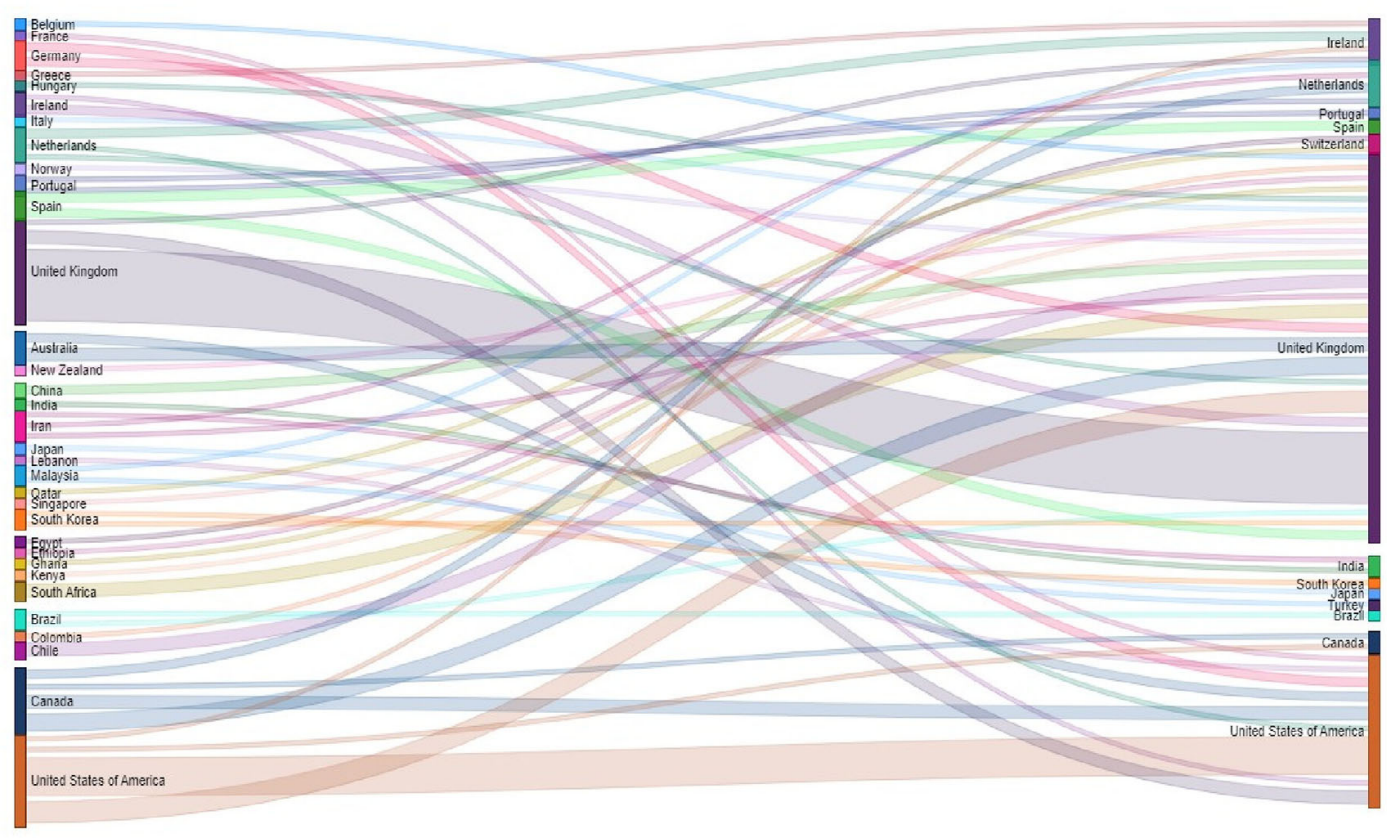
Sankey plot. Country of affiliation of lead author and country of journal of publication.

The correspondence between the country of affiliation of the main author and the country of the journal where the article is published has been visualized by means of the Sankey diagram in Figure 13. It can be observed that nearly 57% of the total publications are published by authors from the United Kingdom, and that authors with a UK affiliation publish 80% of their articles in UK journals, 15% in the United States and only 5% in other countries.

Similarly, authors affiliated to the United States of America publish 56% of their articles in United States journals, 31% in United Kingdom journals, and the remaining 13% in journals of other countries. Although Ireland ranks third in terms of number of journals, authors affiliated to Ireland did not publish in journals of this geographical location, but in the United Kingdom and the United States of America. Authors affiliated to Canada do not publish mostly in Canadian journals either, but 40% publish in UK journals, followed by 30% in the United States, 20% in the Netherlands, and only 10% in Canadian journals.

### 6.5 Institutions and countries

The affiliated entities of the main author that dominate the publication of articles are the universities in a proportion close to 80%, followed by entities of governmental level with 8%, being located hospitals, private companies of investigation and other organizations in the remaining proportions indicated in Table 5.

**Table 5.** Institutions by affiliation of lead author that publish articles related to the research topic.

Institution/entity	Publications/Articles	%
University	82	79.61%
Government Entity	8	7.77%
Hospital institution/research	7	6.80%
Private health research company	4	3.88%
Non-profit organization	2	1.94%
Total	103	

Source: Authors' own elaboration.

**Figure 14.**
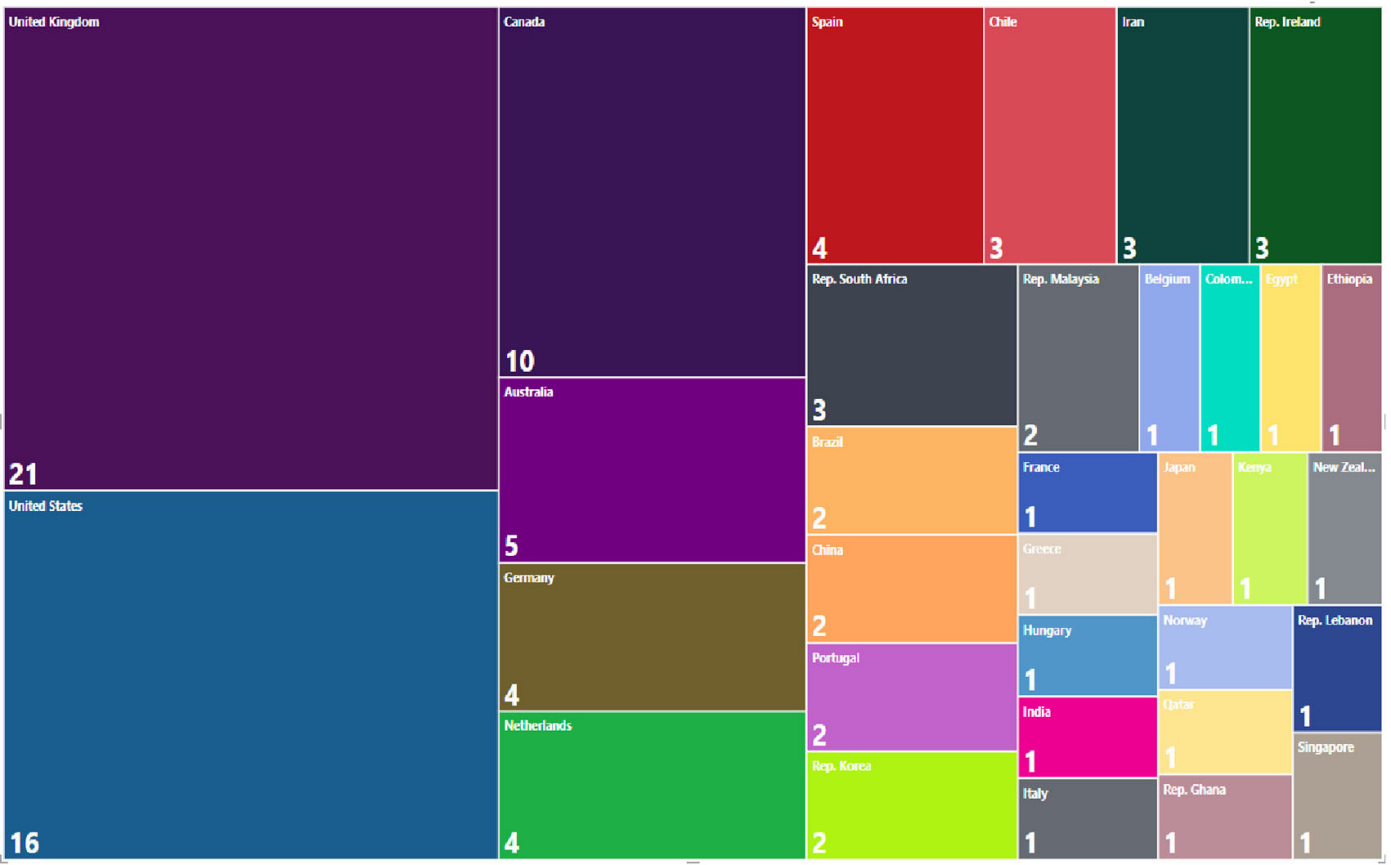
Treemap. Publications by country of affiliation of the main author for the research topic. 1998-2023.

The countries that contribute the most to the generation of evidence according to the affiliation of the lead author are the United Kingdom with 20% of the research corpus, followed by the United States with 16%, Canada with 10% and Australia with 5%, which means that more than 50% of the publications are concentrated in only 4 out of 33 countries. The overall distribution is shown in Figure 14.

**Figure 15.**
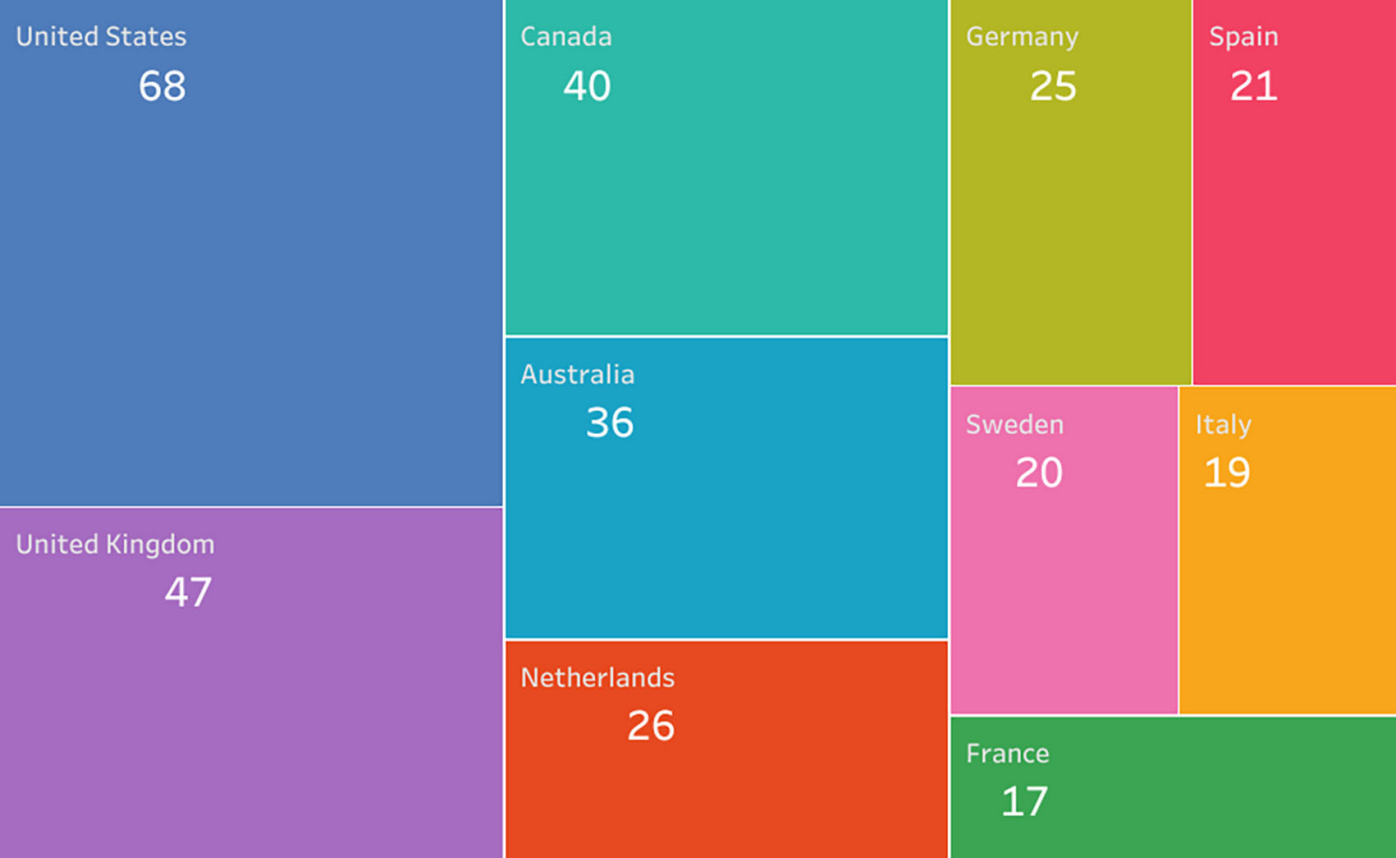
Treemap. Number of primary studies per country according to each SLR. 1998-2023.

We also visualized the countries where the primary studies that included each SLR of the research corpus were developed, finding a total of 826 origins of the studies developed in 132 countries. The origins were defined considering that if an SLR reported 6 primary studies and the 6 studies were developed in the UK, the number of origins was 1, but if the UK was mentioned in two different SLRs, it was counted as 2 origins. In this way, we found that the development environments of primary studies cover wider geographical regions, even if the SLR is published in a particular country. The 10 countries with the highest number of reported studies of these 826 origins are shown in Figure 15 and represent 39% of the total 826 origins.

**Figure 16.**
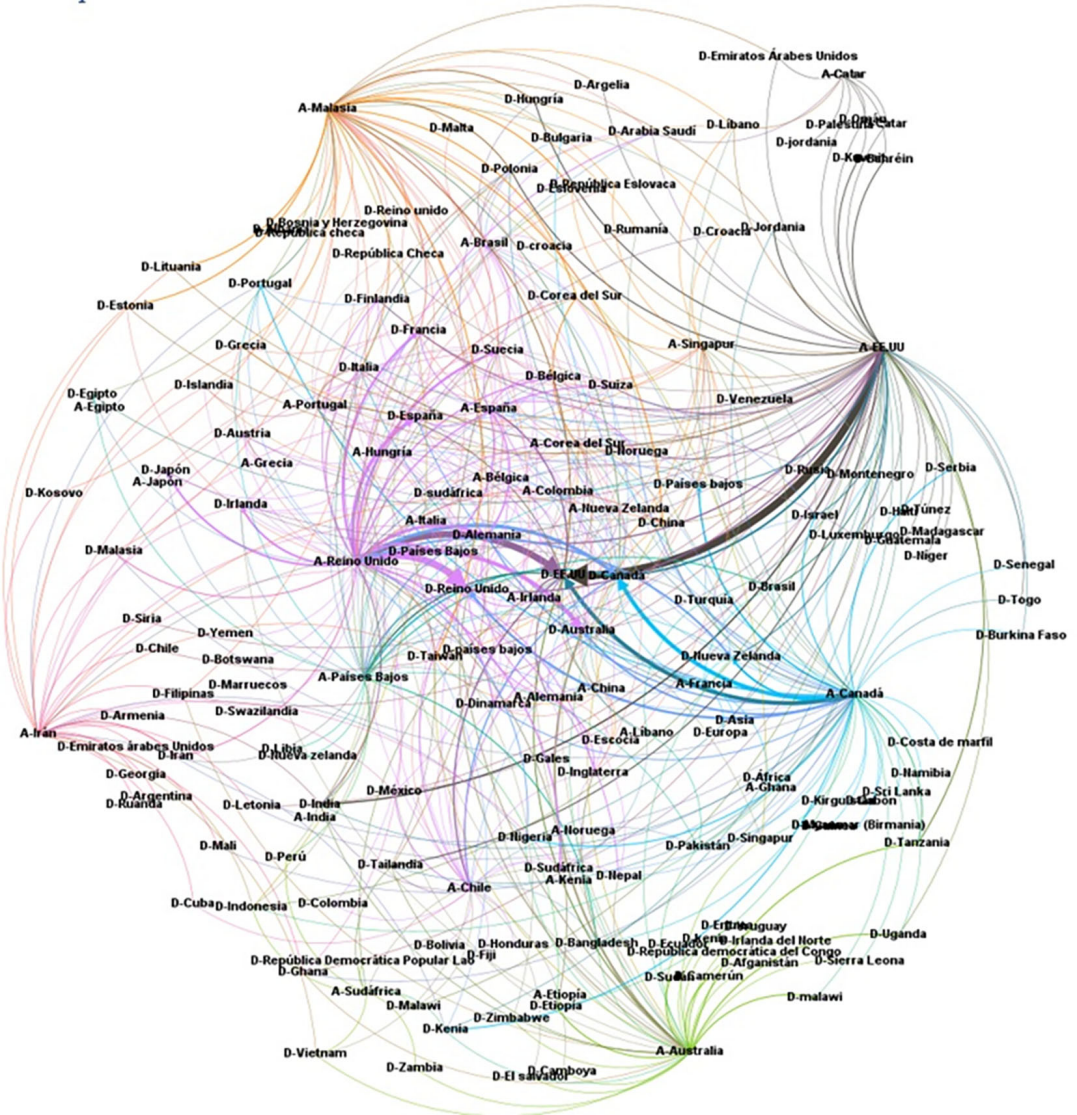
Network of graphs. SLR lead author's country of affiliation and countries of primary studies.

It was considered important to know the relationship between the country of affiliation of the lead author and the country(ies) where the primary studies were developed, thus obtaining the visualization of Figure 16. For example, it can be observed that the SLRs in which the main author reported affiliation to the United States of America (A-USA) include a high proportion of primary studies developed in the USA (D-USA), but also a high proportion of studies developed in other latitudes. The opposite is true if we look at authors with affiliation to Qatar (A-Qatar) in the upper right corner of the graph, who include studies conducted in the same region of West Asia in the SLRs in which they participate as lead author: Bahrain (or Bahrain), United Arab Emirates, Jordan, Kuwait, Lebanon, Oman, Palestine, Qatar, and Saudi Arabia.

**Figure 17.**
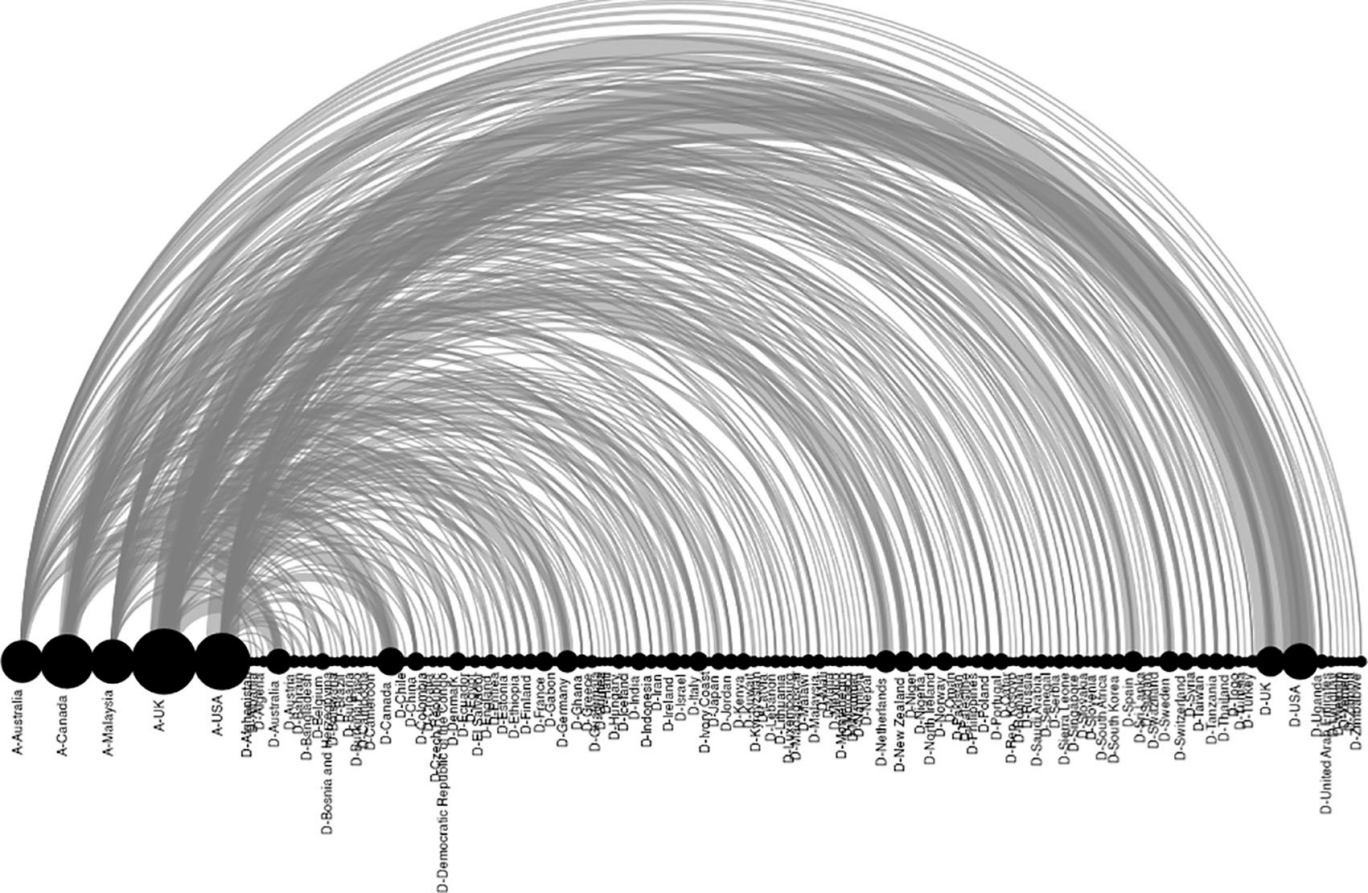
Diagram of arcs by country. Affiliation of the main author (SLR) and primary studies.

The arc diagram in Figure 17 shows the above correspondence in more detail. On the left are the top 5 countries of authors in the SLR that include a greater number of countries of primary studies (starting with A-). These are, in order, the United Kingdom, the United States of America, Canada, Malaysia, and Australia. Below are the countries with the highest proportion of primary studies (starting with D-). Although countries such as the United States of America, the United Kingdom, Canada, Australia and the Netherlands had more primary studies, the diversity of other countries of origin is also observed.

**Figure 18.**
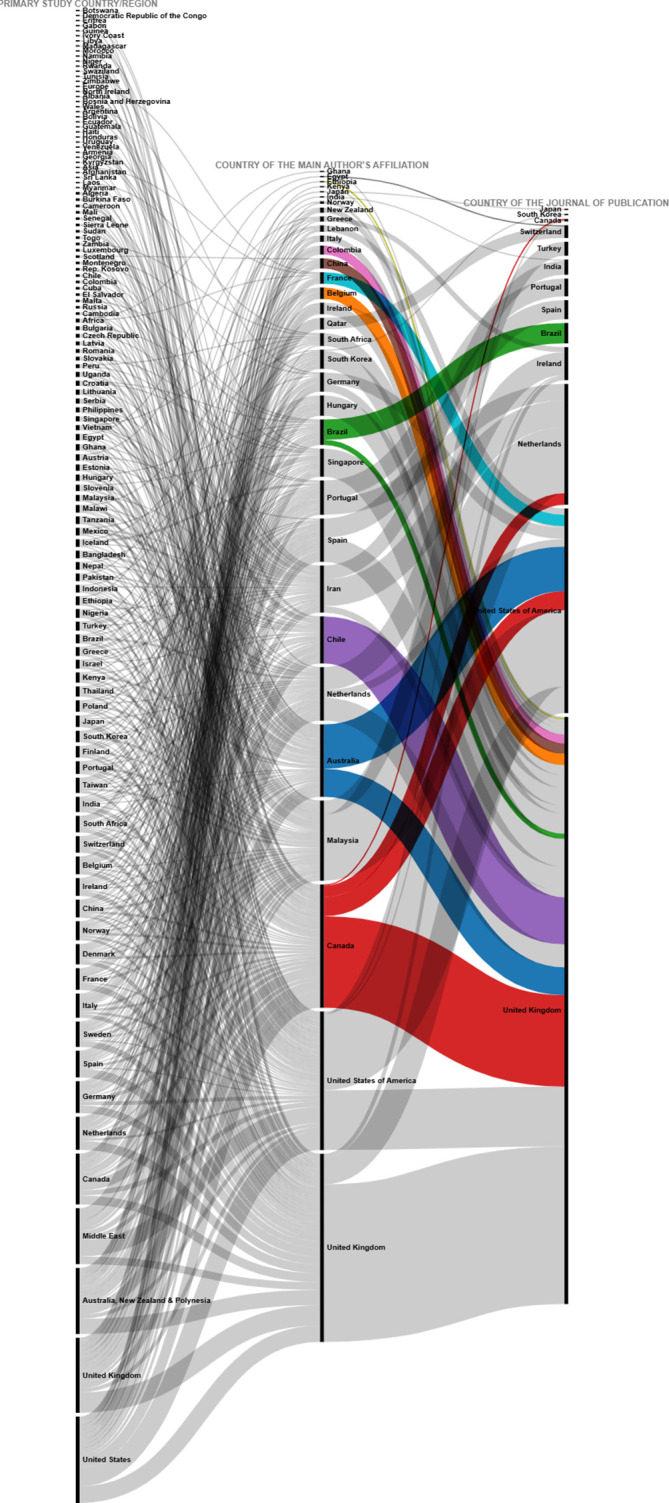
Sankey plot by country: lead study, affiliation of lead author and journal of publication.

To further deepen the analysis, the relationships between the country in which each of the primary studies included in each SLR was conducted, the country of affiliation of the lead author, and the country of the journal in which the SLR was published (13 countries in Figure 6) were visualized using a Sankey diagram, yielding the relationships in Figure 18.

**Figure 19.**
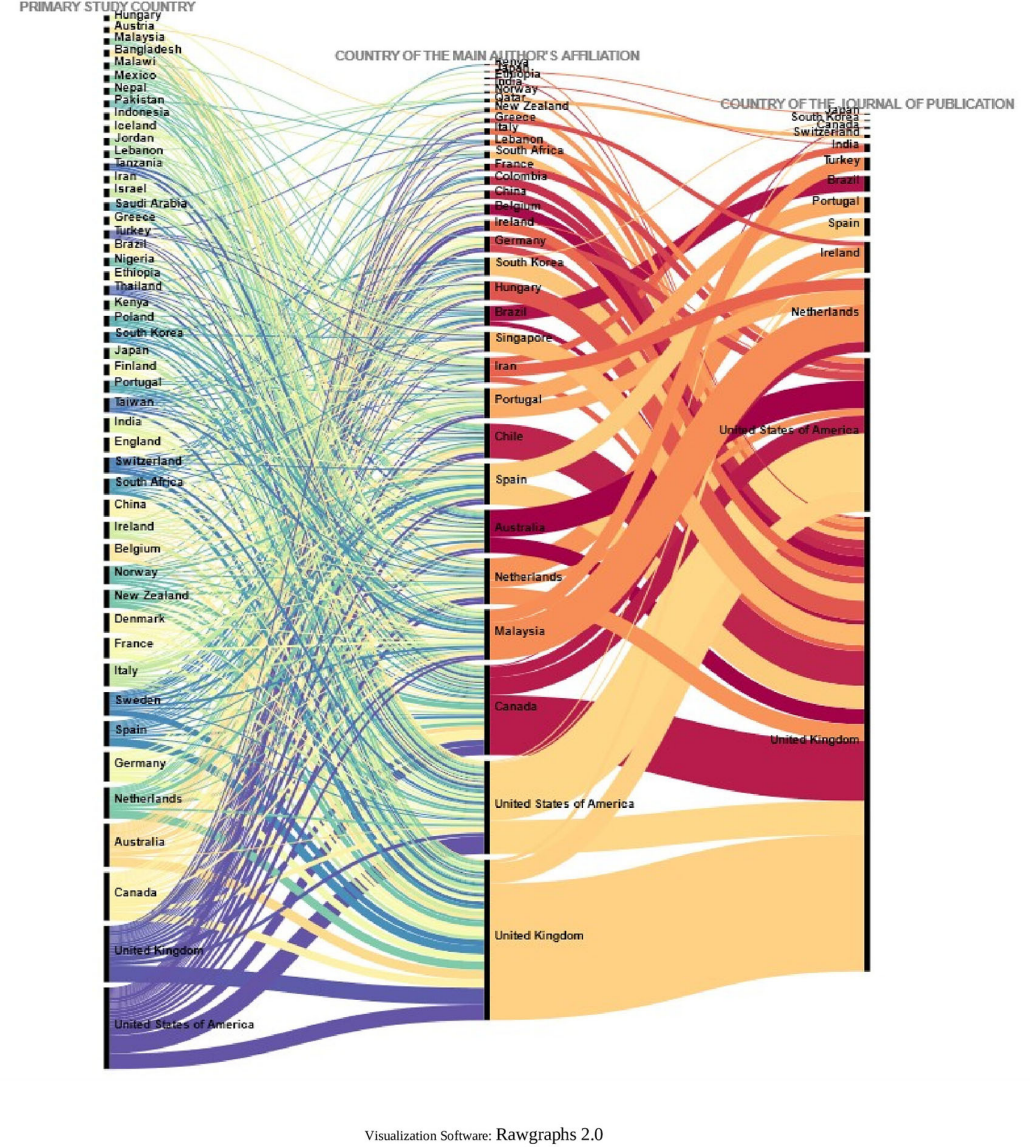
Sankey diagram with details of countries accounting for up to 80% of total primary studies.

To visualize in more detail the behavior shown in the figure above, the same variables were visualized in Figure 19 for the countries where 80% of the primary studies are concentrated, the countries of affiliation of the main authors and the countries of the journals of publication of each SLR. Some interesting behaviors can be observed in this figure, since authors with affiliation in Portugal did not publish their SLR in journals of the United Kingdom and the United States of America, but in Portugal and the Netherlands, and perhaps in Portugal because the SLR included a primary study conducted in Portugal, although no relationship for publication in the Netherlands was identified. Similarly, authors from Malaysia published only in Turkey and the Netherlands, and the SLR published in the Netherlands included a primary study conducted at the same latitude with no link to Turkey.

### 6.6 Funders and sponsors

The information reported in the sponsorship or funding section of each article in the research corpus was extracted, and it was found that 30% of the total 103 SLRs do not include this information. 25% report funding by a government agency at the national level only, and 8% do so by funding a university institution. The remaining 35% are distributed among different organizations or alliances between them ( Table 6).

**Table 6.** Bodies or institutions financing SLR.

Reported study funding	Number of publications/articles	Ratio to total
Not report	31	30.10%
Government Entity	26	25.24%
University	8	7.77%
National collaboration between government entity-University	5	4.85%
Non-governmental entity	4	3.88%
WHO’s international collaboration	3	2.91%
Hospital Research Institute	3	2.91%
Pharmaceutical industry	3	2.91%
WHO international collaboration-Government entity	3	2.91%
International collaboration: University-governmental entity	2	1.94%
Philanthropic Foundation	2	1.94%
International collaboration Universities- ETES Institute- Government entity	2	1.94%
Hospital Research Institute-University	1	0.97%
University-Hospital Research Institute	1	0.97%
WHO-Philanthropic Foundation International Collaboration	1	0.97%
Universities - Philanthropic Foundation	1	0.97%
International Program of Governmental Order	1	0.97%
International collaboration between government entities	1	0.97%
University-Medical Association-Research Institute	1	0.97%
International collaboration University-ETES Agency-government entity Philanthropic Foundation	1	0.97%
University- ETES Agency	1	0.97%
Pharmaceutical industry-government entity	1	0.97%
Government advisory body	1	0.97%
**Total**	**103**	**100.00%**

Source: Authors' own elaboration.

* ETES Agency: Health Technology Assessment Agency.

**Figure 20.**
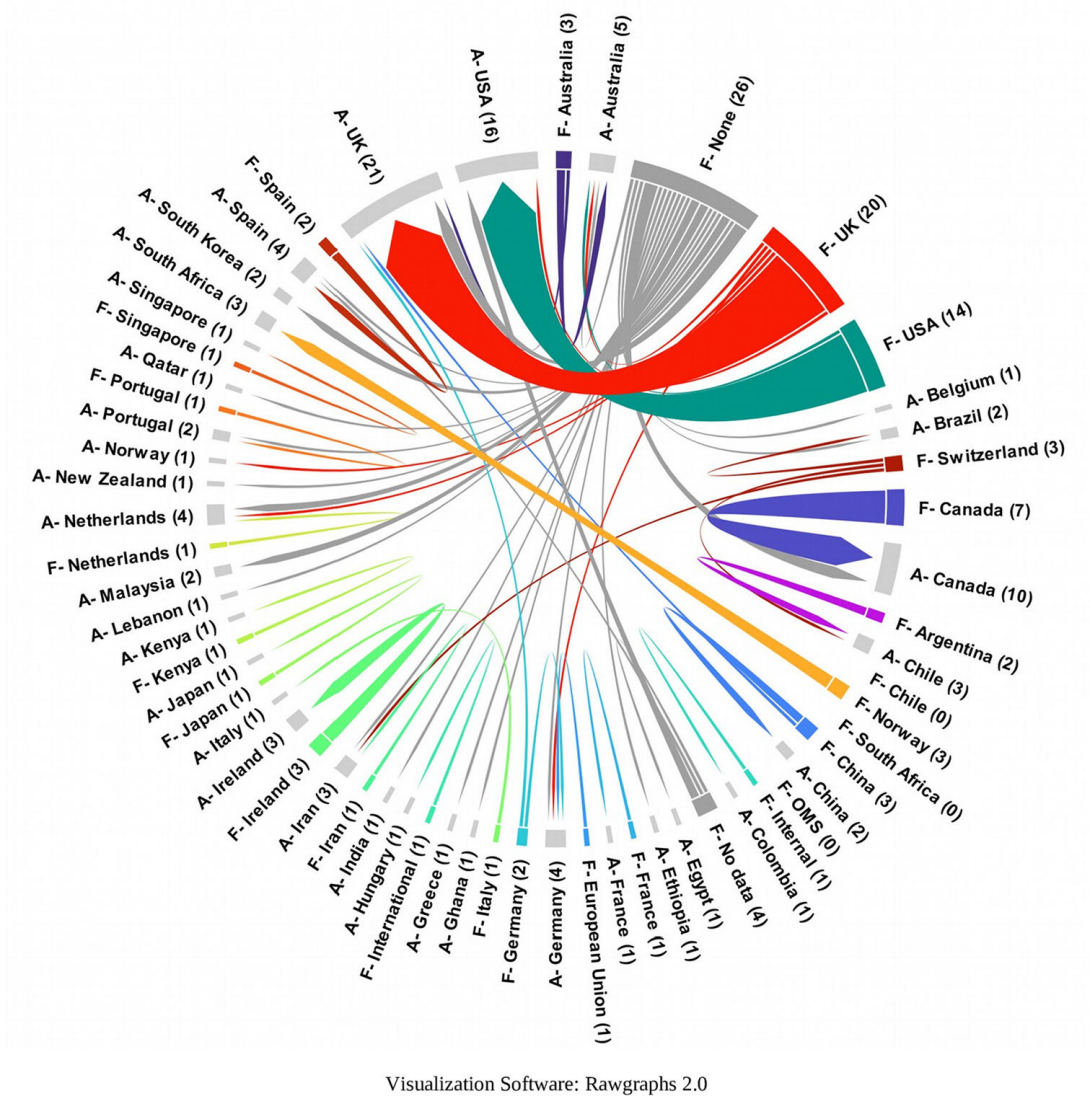
Chord diagram for articles by country of affiliation of lead author and country of funding.

Using a chord diagram to visualize the country of the organization funding each SLR and the country of affiliation of the lead author, it was found that a high proportion of institutions located in countries such as the United Kingdom and the United States of America fund authors with affiliations in the same country. Only a few countries, such as Switzerland, Norway, and China, among others, report funding for authors with affiliations in other countries ( Figure 20).

### 6.7 Cooccurrence of terms

**Figure 21.**
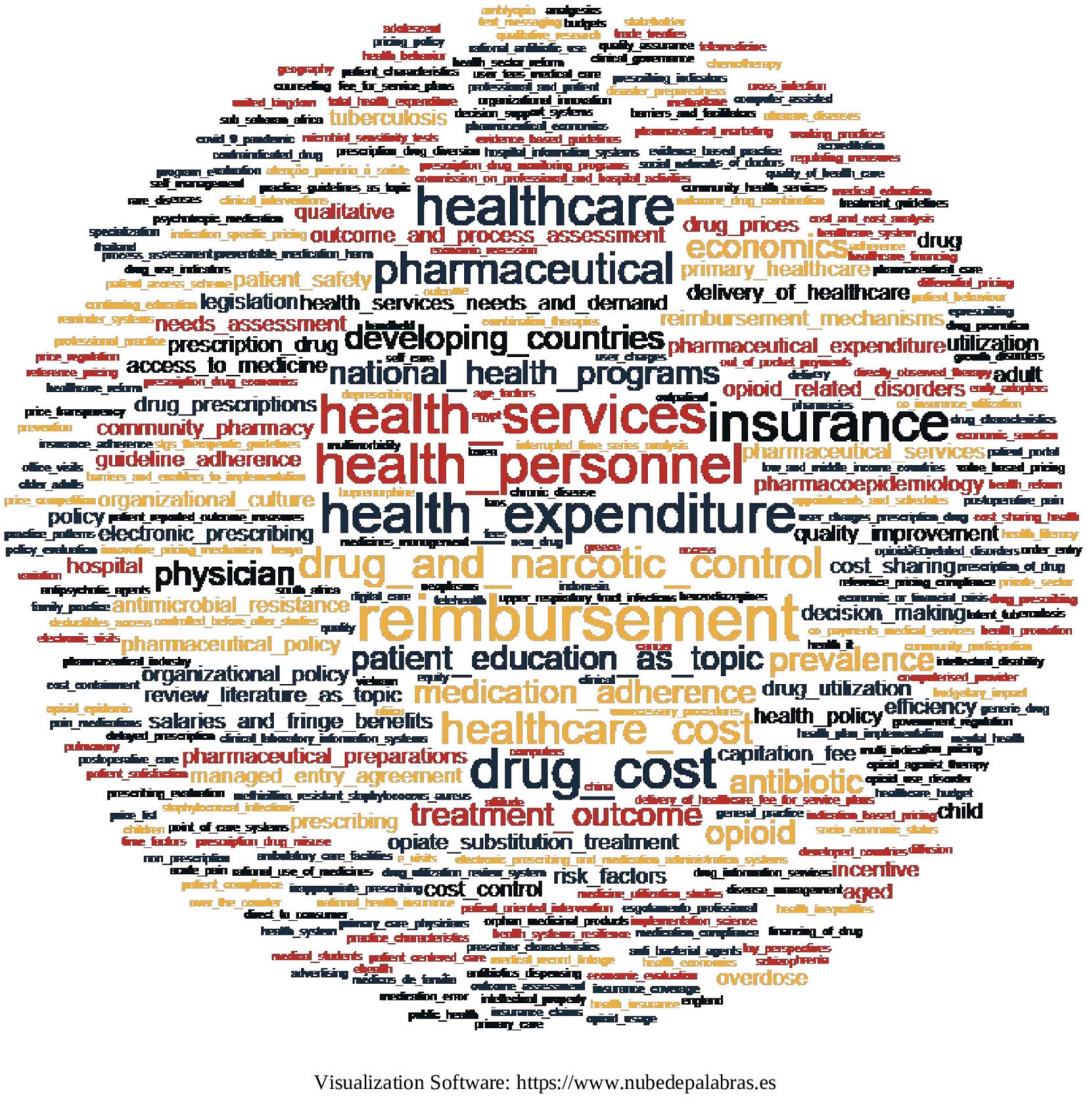
Word cloud of keywords from each article.

Two methods were used to identify the cooccurrence of terms. In the first one, the keywords contained in each article were visualized by means of a word cloud, considering that if the keywords were bigrams or n-grams, they were joined by means of a hyphen under “_” to make them visible in the word cloud in the same way. The result is shown in Figure 21, where the frequency of terms is proportional to the size of the word, with terms predominating:

In the second method, the research corpus was loaded into the Voyant tools to fully capture the terms of each article.

A term cleanup was performed because loading each article includes terms that could skew the results of the word cloud. The cleanup was performed as follows:

a. Plural nouns were changed to singular if the word was previously singular: Drugs-drug.
b. Verbs were recorded as infinitive.
c. Nouns with generic variability and adjectives with two endings were transcribed according to the following rules:
  i. If they appeared only in the feminine form, the feminine form was retained.
  ii. If they appeared only in the masculine form, the masculine form was retained.
  iii. If they appeared in both forms, both are retained.
d. Words related to the type of study were excluded: Randomized-controlled-trials-as-topic, systematic review, etc.
e. All words were converted to lower case.

The n-grams were not concatenated as in the previous word cloud, since the entirety of each article in the research corpus was loaded.

**Figure 22.**
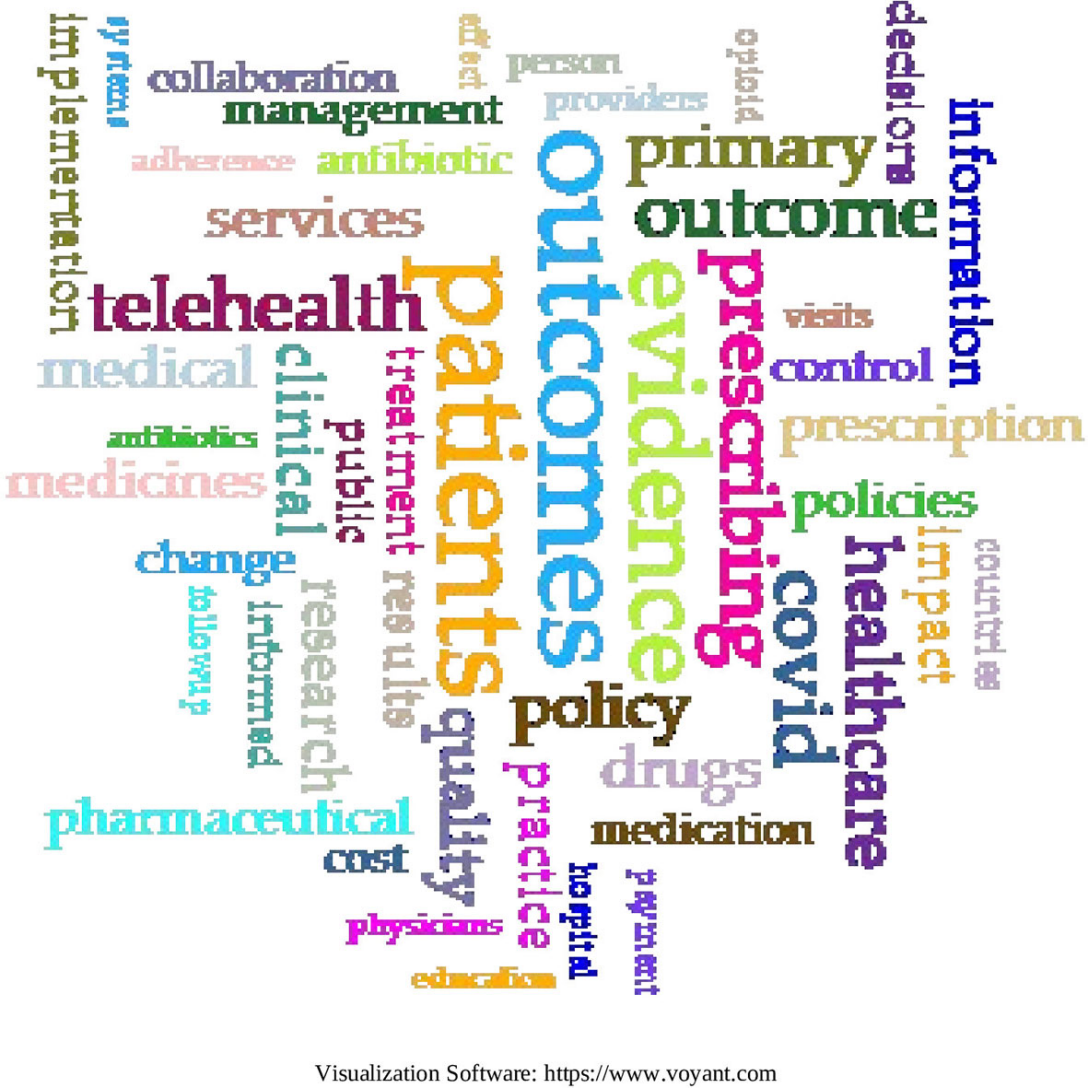
Word cloud to 55 terms from the research corpus.

Common terms in the corpus ( Figure 22):

- Patients (6,260); Outcomes (5,604); Evidence (4,635): prescribing (3,558); COVID (3,355).

### 6.8 Research trends

In order to know the general research trend of each SLR, data were extracted on the type of drugs included in each SLR if they reported it or defined as “general” if they did not mention it. It was found that 50% of the 103 SLRs addressed drugs in their general context, followed by 14% mentioning a focus on antibiotics, 8% on opioids, and 2% on generic and oncology drugs, respectively ( Table 7). These 5 groups represent 77 SLRs out of the total 103. In other cases, the identified groups referred to other drug classifications or several at the same time.

**Table 7.** Type of main medicines on which SLRs are tested.

Type of medicines included in RSLs	Number of publications/articles	Ratio to total	Cumulative
General	51	49.51%	49.51%
Antibiotics	14	13.59%	63.11%
Opioids	8	7.77%	70.87%
Generic Medicines	2	1.94%	72.82%
Oncological	2	1.94%	74.76%
Medicines for non-oncological orphan diseases	1	0.97%	75.73%
Medicines from outpatient programs	1	0.97%	76.70%
Antibiotics, antiretrovirals, and medicines for chronic diseases	1	0.97%	77.67%
Antihypertensives, lipid-lowering agents and antidiabetics	1	0.97%	78.64%
Nervous system and cardiovascular medicines, Antineoplastics and immunomodulators	1	0.97%	79.61%
Antipsychotics	1	0.97%	80.58%
Antidepressants and diabetes	1	0.97%	81.55%
Oral antithrombotics	1	0.97%	82.52%
“NSAIDS COX-2”	1	0.97%	83.50%
Benzodiazepines	1	0.97%	84.47%
Expanded Plan of Immunization Drugs, Antiretrovirals	1	0.97%	85.44%
Benzodiazepines and other psychoactives	1	0.97%	86.41%
Antibiotics, cardiovascular medicines, cocaine, antihypertensives and antidepressants	1	0.97%	87.38%
Prenatal corticosteroids, magnesium sulfate, tocolytics, and antibiotics	1	0.97%	88.35%
Medicines for tuberculosis treatment	1	0.97%	89.32%
Potentially inappropriate medicines	1	0.97%	90.29%
Antibiotics, inhaled costeroids, Innovators and their corresponding generic	1	0.97%	91.26%
Oncological disorders, rheumatoid arthritis, sclerosis	1	0.97%	92.23%
Opioids and medicines for opioid replacement therapy	1	0.97%	93.20%
Statins, antibiotics, opioids, and cardiovascular drugs	1	0.97%	94.17%
Psychotropic	1	0.97%	95.15%
Psychotropic, antihypertensive, hypolipidemic and antidiabetic medicines for heart failure, dementia, antiretrovirals and antibiotics, proton pump inhibitors, warfarin, clopidogrel and benzodiazepines	1	0.97%	96.12%
Quinolones, Antiretrovirals, Hepatitis C, General	1	0.97%	97.09%
Gastric acid suppressants (PPIs and ARH2), NSAIDs, Antipsychotics, Antihypertensives, Statins, COX-2, long-acting opiates and selective serotonin reuptake inhibitors, asthma medications, thiazides and antiplatelets	1	0.97%	98.06%
General and antibiotics	1	0.97%	99.03%
High-cost medicines	1	0.97%	100.00%
**Grand total**	**103**	**100.00%**	

Source: Authors' own elaboration.

**Figure 23.**
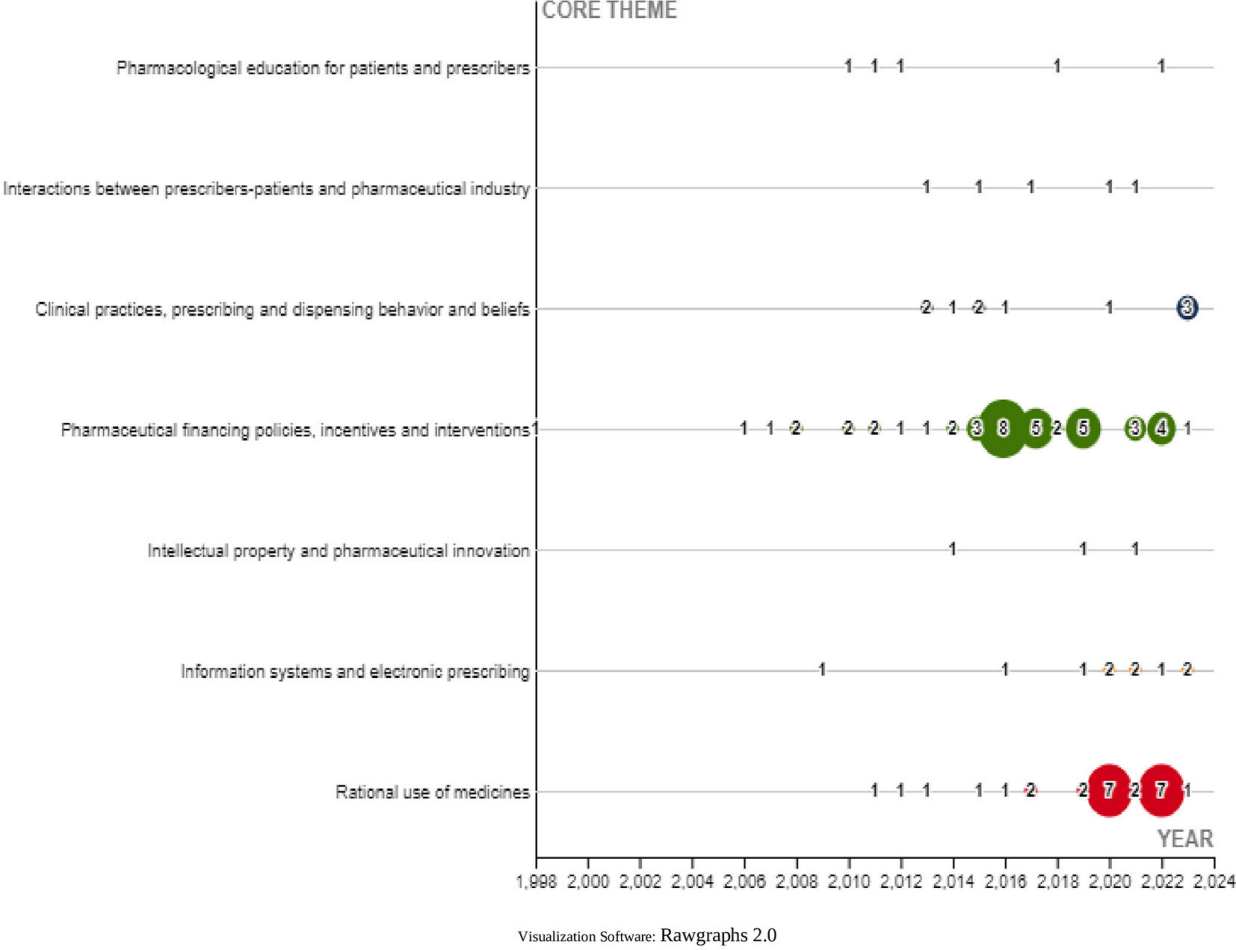
Trends in the central theme on which publications are developed.

Finally, the central theme of each SLR was characterized in a general way and classified into 7 themes described in Figure 23.

## 7. Discussion (Interpretation)

It is observed how the scientometric approach adopted for the mapping has provided a deeper understanding of the characteristics of evidence production on incentives in prescribing and dispensing of medicines and its relationship with pharmaceutical expenditure worldwide, which can be useful in decision making. ^34^

A total of 103 SLRs were analyzed, with the first published in 1998 and an average of less than 5 publications per year. Only until 2015 there is a growth to reach 6 publications in 2022. In recent years, there has been increased production on pharmaceutical expenditure, such as increases in drug prices and procurement, ^35^ the impact on health systems ^36^ or measures to contain it. ^37^ More publications have been observed in recent years, which may be due to more recent publications on public health topics, ^38^ greater innovation in the health field, or simply greater ease of publication and visualization through Internet access in recent years.

When observing the total number of primary studies obtained in the initial search of each SLR of the research corpus, compared to the total number of primary studies included after screening and screening, it was found that less than 1% are retained, a fact produced by the heterogeneity of the studies according to the different approaches proposed by each author, or by methodological quality criteria that are excluded thanks to the rigorous systematic process of the SLR, providing reliable results for our analysis.

By not restricting the search to a single database, those that provide the highest proportion of evidence related to the search topic are identified and may be used to guide future investigations to refine the evidence. Of these, PUBMED and COCHRANE contribute more than 65% of the total SLR.

As for the representativeness of the authors, there is a high dispersion of the authors’ contributions to what Lotka’s law offers a quick panorama that indicates a low productivity, since 94% of the authors appear in a single publication. Only 6% of the total 517 authors (main or co-authors) were in average productivity, so the specialization in the field is possibly low. This fact represents a weakness compared to what has been pointed out by Lassi and Sonnenwald, ^38^ since collaborative work offers advantages for scientific production, among others that papers with several authors can be more cited, in addition to the fact that funders encourage collaborative research, since existing resources can be optimized and also allow the transfer of knowledge and learning. This forces us to think that the research topic is an opportunity for future work.

Oxman A.D., is the author with the highest number of co-authorships, but by the order of signatures, as with Franklin B.D., would appear as director of the research and not as lead author. Ciaponni A. is listed as a co-author in three articles. Perhaps of the few collaborative groups that could be identified are part Grimshaw JM, Peñaloza B, Pantoja T and Herrera CA because the 4 authors are in the same 3 articles.

As for the results of the most cited publications, Horne R. was the author with the highest number of citations, followed by Smith and Shojania K.G. It is necessary to consider that citations do not necessarily correspond to the academic recognition of publications, since for example two of the publications published in 2022 did not submit citations, which can be attributed to the recent nature of the publication. Furthermore, when correlating the authors whose publications have the most citations with the country of the journal in which they are published, the United Kingdom and the United States of America are the leaders.

It should be added that the number of citations may be influenced by other factors such as the title, with informative and attractive titles being more likely to be read and cited. On the other hand, the relationship between title length and citations is ambiguous; in high-impact journals, short titles tend to be more cited, while in other journals, long titles tend to be more cited. The presence of non-alphanumeric characters, such as colons, can increase citations, although the effect varies by discipline. In general, longer articles with more references or more authors tend to receive more citations, as do those with more figures or in English. Oddly enough, making abstracts more readable is often associated with fewer citations. ^39^

To find out which journals contribute most to the scientific production on the research topic, Bradford’s law allowed us to observe the behavior already described by other authors, ^32, 40, 41^ in which a few journals concentrate the majority of the evidence. The 3 core journals that account for 33% of the publications are the Cochrane Database of Systematic Reviews, (UK), PloS One and PloS Medicine of the United States of America, with the same two countries at the forefront of published evidence. Similarly, when the number of publications is related to the geographical region of the journal, the western region of Europe presents the highest proportion of evidence, since the journal CDRS is part of this region and there are other journals, mainly from the British Medical Journal (BMJ).

The impact factor of the journals in which the evidence was published was expected according to the type of publications considered, since as SLR they are usually published in high impact journals, and indeed 40 of the 59 journals were classified in the first quartile (Q1) in Scimago. ^33^ We emphasize that many journals and researchers could focus on this impact factor, which can sometimes be misused for various reasons, ^9^ such as the obsessive search by some universities for recognition associated with publications in journals with a high impact factor or for their researchers to have high research indicators. The impact factor should be indicative and not necessarily related to the quality of the research itself, so that the provisions of the Leiden Manifesto, ^9^ which encourages the consideration of qualitative and quantitative aspects in scientometrics and indicates that quantitative evaluation must support qualitative expert judgement, are of greater importance. ^42^

Leading countries in the publication of evidence of the research topic maintain a similar behavior to that discussed previously according to journals of publication, since in Europe it leads the United Kingdom and in America, United States of America and by relating it to the country of affiliation of the main author and the country of the publication journal, the panorama, although similar, provides additional information because authors from different countries publish mostly in journals of the United Kingdom and then in journals of the United States of America. A very small proportion of authors affiliated to institutions in the United Kingdom and the United States of America publish in journals outside their respective countries. The cause of this behavior could be inferred from a low proportion of “scientific recognition” journals, expressed, for example, as the impact factor in the countries of original affiliation of the authors, which could lead them to look for other countries of publication, a hypothesis that is supported by looking at the countries of publication of journals in which, for example, only one Latin American country appears: Brazil. This behavior has been called “parachute science” or scientific colonialism, which, due to paternalistic practices, sometimes dictates the research agendas of countries with fewer resources. ^43^

Scientific colonialism is linked to modernity, globalization and this epistemic dependence on countries considered central or developed, under which models and approaches focused on the topics that in these countries can be considered relevant to research must be imposed, unable in this way to overcome the internal difficulties to position themselves from their research origin. ^44, 45^

For these reasons, it is necessary to promote research and the publication of results in other countries, including local researchers who develop their research in their context, under the conditions, environments and resources available, and thus respond to the needs they have there.

Publication in countries with a tradition of generating evidence may have a cause in the type of institutions of affiliation of the author, since it is observed that about 80% of publications originate from universities and only 8% from government agencies. Therefore, it is possible that authors may decide to send their manuscripts to other countries because of studies in universities with high academic recognition or because of the desire to obtain intellectual recognition and research prestige.

Moreover, to further deepen scientific colonialism, when evaluating the behavior between the country where the primary studies are developed and the country of affiliation of the main author, it is evident that the countries leading the publications include in their SLR a large number of countries of origin of the primary studies, many of them in developing countries, which is in line with research on the subject focused on colonialism in global health research, which causes local researchers of the research environments with first-hand knowledge in each country to have a limited influence on research. ^46^ In this analysis, it is observed that they continue to lead the United Kingdom and the United States of America, followed by Canada and Australia, but new actors enter such as Malaysia, which offers a different behavior because it includes in its SLR, primary studies of the same environment in the Asian region mostly and to a lesser extent, primary studies in countries such as the United Kingdom and the United States of America (see Figure 16).

The funnel or concentration effect, which is shown by relating the country of the primary study included in the SLR, the country of main affiliation and the country of the publishing journal, shows how authors from the United Kingdom or the United States of America publish in journals from these same countries. This may be due to the increased availability of resources in these countries, which may result in funding that requires studies to be published in journals in the same country. The “advantage” offered by journals based mostly in high-income countries, as opposed to the benefit they provide, has been discussed in the analysis of how authors are increasingly required to publish in open access journals, with the associated article processing fees, and the possible exclusion of authors from low-income countries due to lack of resources. ^46^

A few cases do not follow scientific colonialism, as authors from Malaysia and Portugal published their SLRs in Turkey and the Netherlands in the first case, and in the Netherlands and Portugal in the second case, possibly because some SLRs included primary studies conducted in the same country of publication, which could make it easier for the journal to accept each article.

The implications of this “scientific colonialism” in health-related publications can lead to important phenomena that should be considered by governments, especially in low- and middle-income countries, such as inequalities in knowledge production, because most health research is generated in high-income countries, creating a bias in available knowledge ^47^ and potentially leading to the neglect or misinterpretation of the health priorities of communities in low- and middle-income countries.

Similarly, there are failures of representation and influence in scientific discourse and global health policy, as researchers from non-Western countries face difficulties in accessing high-impact publishing platforms. ^48^ Thus, research conducted in developing country contexts tends to unequally benefit institutions in developed countries where findings are disseminated. ^49^ This means that local communities may not directly benefit from research that uses their resources.

On the other hand, there may be a devaluation of local knowledge, such as traditional health knowledge and indigenous knowledge, which may be undervalued in different contexts, leading to interventions that are neither culturally appropriate nor effective. ^50^

Often, research in low- and middle-income country contexts does not adhere to the same ethical standards observed in developed countries, including a lack of or asymmetries in adequate informed consent. ^51^

Finally, difficulties may arise in implementing findings, as recommendations formulated in Western contexts may lack applicability or sustainability in local contexts, limiting the effectiveness of health interventions. ^52^

For these reasons, it is critical to promote equitable collaborations, value and respect local knowledge, and ensure that communities in low- and middle-income countries benefit from research.

The likelihood of greater availability of resources in some countries is greater according to other results of this research, for example when the funding is visualized, because despite the fact that in 30% of the SLR do not report a specific source, 70% do, being in this proportion the majority exclusively governmental and national (25.25%), or in collaboration with other national or international entities or governments (15, 53%).

These proportions of funding may be due to the fact that governments in high-resource countries have more money to spend on research, which means that researchers affiliated with universities and research institutions apply for grants and may develop their studies to fit the specific doctrines and themes of global health funders, rather than the other way around. ^46^ Only a few high-income countries, such as Switzerland, Norway, or China, were found to fund researchers with external affiliations.

It is important to note that 30% of SLRs do not report or do not receive funding, as this could be interpreted as greater independence in the judgments made, as evidence has been found that the private sector, through more direct actions such as funding researchers and universities, could threaten the independence of research. Some authors ^53, 54^ point out how the funding of private companies can negatively influence the results of research, as they may come to condition not only what is studied, but also its results.

In this research, 479 keywords were found in the 103 SLRs that were included in 67 SLRs of the research corpus. In order to know the emerging trends in the generation of evidence, we resorted to the analysis of the cooccurrence of words and found that the keywords with the highest cooccurrence are related to the central topics on which the research is focused and quickly address the trend in the generation of evidence. The results show a concentration on topics such as reimbursement, insurance, drug costs, healthcare services, healthcare personnel and, of course, healthcare spending. This confirms that knowledge and analysis of health expenditure and its financing are issues of great importance, not only for their impact on the financial sustainability of any health system, but also for the access that the population must effectively achieve, ^55^ therefore, it is congruent that the articles included in this research mention these aspects.

In the second co-occurrence analysis on the total terms of the 103 SLRs, a total of 2,023,091 words and 78,139 unique word forms were obtained. The co-occurrence words most often refer to more specific topics, since the entire text of each article is used, and thus they appeared: Patients, Results, Evidence, Prescription and COVID; the first 4 closely related to the concepts found in the analysis by keywords and the fifth concept of COVID, appears possibly due to the avalanche of evidence that was generated related to this topic after the 2020 pandemic and that in that year reported an exponential rate of global publication around 500 publications daily, ^56^ so it is logical that this topic affects searches that include this period as it is a new factor that enters the global pharmaceutical spending.

When analyzing the types of drugs studied in the research corpus, about 50% of the research is found without a specific group of drugs, which suggests that it is a global issue that could then be focused on some specific types, such as antibiotics, which are a central and unique topic in 13% of the 103 SLR, possibly due to the global concern related to their rational use and microbial resistance generated by poor practices in prescribing and dispensing. ^57^ In a smaller proportion, the SLRs whose main topic is the use of opioids, a topic of global concern on which the World Health Organization issues constant recommendations ^58^ and which in countries such as the United States of America has generated what has been called the opioid epidemic. ^59, 60^

Finally, among the topics characterized in the research corpus, the most visible are those related to financing policies, incentives and pharmaceutical interventions followed by rational use of medicines. These topics have been discussed by other researchers in the analysis of drug financing in health systems, ^61^ including pricing policies, financing of new drugs ^62^ and analysis of the impact that policies can have on prescribers as one of the main actors of health systems. ^63^

## 8. Conclusions

This research work achieves the fulfillment of the main objective to explore the panorama of the global scientific production of open access articles, related to the prescription and dispensing of medicines and its relationship with the pharmaceutical expenditure faced by health systems.

In this sense, the use of scientometric mapping allowed to know the trends in the generation of evidence related to the research topic, identifying the growth in annual publications since 1998, accentuated in recent years possibly by the impact that the prescription and dispensing of medicines and the consequent pharmaceutical expenditure on the sustainability of global health systems. It also allowed to visualize the most representative authors, the most cited research and publication trends according to the origin of the journal, the affiliation of the main author and the environments where the primary studies of each SLR were developed.

In general, there is a marked influence on the global scientific production on the topic of research represented by countries such as the United Kingdom and the United States, with more publications in journals and studies from these countries. The main research topics were financing policies, pharmaceutical incentives and interventions, and rational use of medicines. A scarce and worrying generation of evidence was observed mainly at the Latin American level, which may be an interesting topic for future research. As more studies from other countries can be included or developed, the results can be evaluated in different latitudes, thus contributing to the development of evidence and policies that support the sustainability of health systems and patients’ access to medicines.

## 9. Strengths and limitations

Although the results of this research are informative, some limitations inherent to the scientometric methods used are reported. First, although the search method included a large number of open access databases, which is considered a strength in terms of reproducibility and transparency of the search, the publication search algorithm specifies a restriction on identified keywords that must be present in titles or abstracts, and although it has advantages in refining the selection process, it may exclude relevant articles that are only available in restricted access or that use other keywords to refer to the research topic. Therefore, it is interesting to consider the use of other keywords and searches in paid databases to conclude whether the pattern of results is maintained or whether there are differences when using open access and restricted access information.

Second, as observed in the analysis of annual publications, since this is an evolving field of research, we may have underestimated the contribution of other recently published documents because they are not included as SLR.

However, even with these limitations, this scientometric mapping has resulted in a broad and important global view of the research topic and its state of the art to date.

Finally, it is important to recognize that, despite the findings presented, there are biases that may affect the results. In particular, selection bias and inherent bias in systematic reviews of the literature, which are derived from the primary studies analyzed, may significantly affect the validity of our conclusions. Therefore, it is necessary to consider these factors with caution when interpreting the results, as they could affect the generalizability of our findings and distort certain relationships, either underestimating or overestimating them. Therefore, we recommend that future studies focus on addressing these biases to provide a more accurate and complete picture of the phenomenon in question.

## Ethical approval

This study was conducted using data retrieved from scientific evidence databases. Due to no human subjects’ involvement, ethical approval was not required.

## Authors’ contributions

Conceptualization: LT, GJ, JR. Methodology: LT, GJ, JR. bibliographic search and data analysis: LT. Visualization: LT. Writing – Original Draft Preparation: LT. Writing - Review & editing: LT, GJ, JR. Supervision: GJ, JR.

## Supplementary data

There is no supplementary material in addition to that included under “data availability”.

## Data Availability

The data associated with this article are presented in the corresponding supplementary data, located at the following link Repository name: Figshare. Checklist for “Incentives in prescribing, dispensing and pharmaceutical spending: A scientometric mapping”. DOI: https://doi.org/10.6084/m9.figshare.26948281.v2 ^64^ *This project contains the following underlying data:* Luis Tocaruncho-Supplementary Material.docx. Dataset Data are available under the terms of the Creative Commons Zero “No rights reserved” data waiver (CC0 Public domain dedication). For further information, please contact the authors.
