# Supplementary material for: Incentives in prescribing, dispensing and pharmaceutical spending: A scientometric mapping
Source: F1000Res. 2025 Feb 11;13:1333. Originally published 2024 Nov 7. [Version 2] doi: 10.12688/f1000research.156306.2 (PMC11826077; doi:10.12688/f1000research.156306.2)
Supplement: Peer review history

# Reviewer report: Reviewer response for version 2

**DOI:** 10.5256/f1000research.177628.r365736

**Published:** 2025-02-13

**Authors:** Mosab Hammoudeh

**Affiliations:** 1 University of New Orleans, New Orleans, USA

The authors have adequately addressed my initial comments.

Is the work clearly and accurately presented and does it cite the current literature?

Partly

If applicable, is the statistical analysis and its interpretation appropriate?

Partly

Are all the source data underlying the results available to ensure full reproducibility?

Yes

Is the study design appropriate and is the work technically sound?

Partly

Are the conclusions drawn adequately supported by the results?

Partly

Are sufficient details of methods and analysis provided to allow replication by others?

Yes

Reviewer Expertise:

Corporate finance; pharmaceutics and economics; product markets; corporate innovation; mergers and acquisitions

I confirm that I have read this submission and believe that I have an appropriate level of expertise to confirm that it is of an acceptable scientific standard.

# Reviewer report: Reviewer response for version 2

**DOI:** 10.5256/f1000research.177628.r365737

**Published:** 2025-02-13

**Authors:** Churchill Akena

**Affiliations:** 1 Pharmacy, Makerere University School of Health Sciences (Ringgold ID: 373383), Kampala, Central Region, Uganda

Having gone through the revised article, I have found no need for any further comments. Thank you

Is the work clearly and accurately presented and does it cite the current literature?

Yes

If applicable, is the statistical analysis and its interpretation appropriate?

Yes

Are all the source data underlying the results available to ensure full reproducibility?

Yes

Is the study design appropriate and is the work technically sound?

Yes

Are the conclusions drawn adequately supported by the results?

Yes

Are sufficient details of methods and analysis provided to allow replication by others?

Yes

Reviewer Expertise:

Pharmaceutical science, public health

I confirm that I have read this submission and believe that I have an appropriate level of expertise to confirm that it is of an acceptable scientific standard.

# Reviewer report: Reviewer response for version 1

**DOI:** 10.5256/f1000research.171596.r352096

**Published:** 2025-01-15

**Authors:** Mosab Hammoudeh

**Affiliations:** 1 University of New Orleans, New Orleans, USA

The authors of this paper employ scientometric mapping to analyze global scientific open-access literature on prescribing and dispensing practices and their relationship with pharmaceutical expenditures in health systems. The article analyzes 103 systematic literature reviews, then provides illustrations that summarize statistics related to articles, journals, databases, key contributors, citations, institutions, countries, funders, research trends, and drug types. The United Kingdom and the United States dominate this scientific production, while Latin America, Africa and Asia show limited contributions. This last finding is attributed to the scarcity of high-impact journals in other regions. The authors introduce the term “parachute science” or “scientific colonialism” wherein researchers affiliated to institutions in leading countries publish studies conducted in other regions. They then recommend the involvement of local authors in research projects, which may better address local needs. The study provides insights for policymakers and researchers on the dynamics of pharmaceutical spending and related evidence generation.

 The article has several strengths. First, it follows a systematic approach with clearly defined stages (retrieval, migration, analysis, visualization, and interpretation) which improves transparency and replicability. Second, it provides a comprehensive view of the open-access literature by including 103 SLRs spanning multiple databases and countries. Third, it provides accessible representations of complex scientometric data by utilizing visualization software. Finally, the article highlights the underrepresentation of Latin America and other regions in the literature and provides actionable insights for future research.

 Therefore, my decision for the approval status of this article is Approved with Reservations. The following is a discussion for some of the areas in which the article can be improved:

1. Representativeness of the sample articles. While the authors collect their articles from multiple databases, the question remains on whether open-access databases are a representative sample for the true population of articles. To illustrate this point further, I have added Table 1 below which includes statistics on pharmaceutical drug development by country. As shown in the table below, the distribution of the most productive countries by pharmaceutical drug development is similar to the distribution of the most productive countries by research publications found in this article, but with some significant differences. For example, the US is by far the most productive country by drug development where 54% of all drugs are developed within the US, whereas the UK only accounts for about 9% of all drugs developed. Moreover, China, Japan and South Korea are among the most productive drug developers, whereas they appear to have very low article publication productivity. These differences (between the distribution of countries by drug development and by research productivity) may be attributed to limitations in open-access databases. To address this comment, the authors should include a discussion on the representativeness of open-access databases and how they compare to other databases.
2. The authors emphasize several times that this paper aims to examine the relationship of the drug prescription and dispensing literature with pharmaceutical expenditures in health systems worldwide, yet little evidence was presented directly relating to pharmaceutical expenditures, with the exception of Table 6 and Figure 21. The authors may consider rewording the introduction and study objectives to more accurately reflect the contribution of this paper, which in essence is an exploratory study that provides a detailed account for different characteristics of global pharmaceutical SLR.
3. Limited Analysis Depth: While the scientometric mapping identifies trends, the analysis could benefit from deeper exploration of the implications of these trends on healthcare policies and practices. In otherwords, the focus on general trends without delving into specific interventions or outcomes limits its practical applicability for policymakers. For example, the authors discussed scientific colonialism, but did not provide any additional evidence that highlights the harm or shortcomings related to such an issue.
4. Cross-Disciplinary Insights: The articles were retrieved from databases with a medical focus. Drawing connections with disciplines like economics or behavioral science could provide a richer understanding of the factors influencing prescribing behaviors.
5. Many of the figures are too cluttered and are illegible (These are figures 2, 6, 10, 16, 17, 18 and 19). The figures are impossible to read without zooming in, and once zoomed in, the quality of the image deteriorates significantly. This is a serious problem that renders these figures as valueless. The authors should consider rethinking how they want to present these figures so that they are more legible. Here are some suggestions:
  1. Consider displaying a smaller number of the most important items and dropping the rest. The author already do this in several of the figures, however, they should include only the legible figure and drop the cluttered one, e.g., keep Figure 6 and drop Figure 7, keep figure 23 and drop figure 22.
  2. Consider lumping countries in the same region together to reduce the number of label, especially for countries that are less productive. For example, Jordan, Lebanon, Qatar, UAE and KSA can all be labeled as Middle East (or ME) which could help make the figures less cluttered.
6. Correcting Potential errors: Looking up the single article included from 1998 (Zara Yahni, 1998), the article cites 43 papers, however, Table 1 mentions that 34 total studies were included in the RSL (which I assume is a typo for SLR).

Is the work clearly and accurately presented and does it cite the current literature?

Partly

If applicable, is the statistical analysis and its interpretation appropriate?

Partly

Are all the source data underlying the results available to ensure full reproducibility?

Yes

Is the study design appropriate and is the work technically sound?

Partly

Are the conclusions drawn adequately supported by the results?

Partly

Are sufficient details of methods and analysis provided to allow replication by others?

Yes

Reviewer Expertise:

Corporate finance; pharmaceutics and economics; product markets; corporate innovation; mergers and acquisitions

I confirm that I have read this submission and believe that I have an appropriate level of expertise to confirm that it is of an acceptable scientific standard, however I have significant reservations, as outlined above.

# Response

**Published:** 2025-01-28

**Authors:** Luis Hernando Tocaruncho Ariza

**Affiliations:** University of Bogota Jorge Tadeo Lozano Faculty of Natural Sciences and Engineering, Bogotá, Bogota, Colombia

Dear Mosab Hammoudeh,

 We are very grateful for your invitation to resubmit a revised version of the manuscript and also appreciate each of your constructive comments. The manuscript has been revised and improved in response to your comments. Additional changes have been made based on further reading and discussion.

 In the following,  we address each of the issues raised and summarize the changes made to the manuscript, and we hope to meet your expectations and thank you again for your advice, which was extremely useful to give more scientific rigor to our manuscript.

*Reviewer Comment: Representativeness of the sample articles. While the authors collect their articles from multiple databases, the question remains on whether open-access databases are a representative sample for the true population of articles. To illustrate this point further, I have added Table 1 below which includes statistics on pharmaceutical drug development by country. As shown in the table below, the distribution of the most productive countries by pharmaceutical drug development is similar to the distribution of the most productive countries by research publications found in this article, but with some significant differences. For example, the US is by far the most productive country by drug development where 54% of all drugs are developed within the US, whereas the UK only accounts for about 9% of all drugs developed. Moreover, China, Japan and South Korea are among the most productive drug developers, whereas they appear to have very low article publication productivity. These differences (between the distribution of countries by drug development and by research productivity) may be attributed to limitations in open-access databases. To address this comment, the authors should include a discussion on the representativeness of open-access databases and how they compare to other databases.*

 Following your recommendation, we conducted a through analysis of the representativeness of open access databases compared to other databases and found that they offer significant advantages in providing greater clarity and context to the manuscript. Accordingly, we have conducted a search for related evidence and include an analysis in the introduction of the manuscript, in which we aim to make clear the factors that may affect the representativeness of open access databases versus other databases.

 We are deeply grateful for your detailed review and valuable recommendations. We are convinced that your comments have contributed significantly to improving the revised version of our manuscript.

***Reviewer Comment:** The authors emphasize several times that this paper aims to examine the relationship of the drug prescription and dispensing literature with pharmaceutical expenditures in health systems worldwide, yet little evidence was presented directly relating to pharmaceutical expenditures, with the exception of Table 6 and Figure 21. The authors may consider rewording the introduction and study objectives to more accurately reflect the contribution of this paper, which in essence is an exploratory study that provides a detailed account for different characteristics of global pharmaceutical SLR.*

 We agree with and appreciate your suggestion and have therefore reformulated the main objective of the review, clarifying in the Introduction and Methods section that the review is based on an exploratory design that provides a detailed description of the characteristic panorama of the global scientific production of Open Access articles in relation to the prescription, dispensing and pharmaceutical expenditure faced by health systems. In this way, we focus the reader on the scientometric results of our review and limit the scope of the review.

***Reviewer Comment:** Limited Analysis Depth: While the scientometric mapping identifies trends, the analysis could benefit from deeper exploration of the implications of these trends on healthcare policies and practices. In otherwords, the focus on general trends without delving into specific interventions or outcomes limits its practical applicability for policymakers. For example, the authors discussed scientific colonialism, but did not provide any additional evidence that highlights the harm or shortcomings related to such an issue.*

 We are very grateful for your highly analytical comments and for recognizing the importance of deepening the discussion of scientific colonialism in our manuscript, therefore we have included in the discussion section, an analysis through a search of related references, thus finding your suggestion essential to discuss this key element and refine the accuracy and understanding in this regard.

***Reviewer Comment**: Cross-Disciplinary Insights: The articles were retrieved from databases with a medical focus. Drawing connections with disciplines like economics or behavioral science could provide a richer understanding of the factors influencing prescribing behaviors.*

 As you rightly indicate, and according to the adjustments made thanks to your valuable suggestions, we have defined that the objective of this article is: to explore and provide a detailed description of the characteristics panorama of the global scientific production of Open Access articles related to the prescription, dispensing and pharmaceutical expenditure faced by health systems. In the introduction it is mentioned:

 “The results of this scientometric mapping that visualizes scientific production will be complemented in a second research with a synthesis of evidence focused on the map review to analyze the trend of scientific production identified, ^8^ and is part of a doctoral research focused on identifying and analyzing the factors that influence the cost of prescribing, dispensing and recognition costs of medicines in the Colombian health system from performance information from 2020 to 2022.”

 This second article, which will identify and analyze in detail the factors that influence prescribing behavior, is in the submission stage, so when it is finalized we will be happy to send you a copy for your valuable comments.

***Reviewer Comment: **Many of the figures are too cluttered and are illegible (These are figures 2, 6, 10, 16, 17, 18 and 19). The figures are impossible to read without zooming in, and once zoomed in, the quality of the image deteriorates significantly. This is a serious problem that renders these figures as valueless. The authors should consider rethinking how they want to present these figures so that they are more legible. Here are some suggestions:*

1. *Consider displaying a smaller number of the most important items and dropping the rest. The author already do this in several of the figures, however, they should include only the legible figure and drop the cluttered one, e.g., keep Figure 6 and drop Figure 7, keep figure 23 and drop figure 22.*
2. *Consider lumping countries in the same region together to reduce the number of label, especially for countries that are less productive. For example, Jordan, Lebanon, Qatar, UAE and KSA can all be labeled as Middle East (or ME) which could help make the figures less cluttered.5. *

Your detailed analysis and feedback has been crucial to optimize the presentation and display of our manuscript. To address these problems, and following your suggestions, we have modified Figure 2, Figure 6 was removed, Figure 10 (now Figure 9) was modified restricted to journals with 2 or more publications, Figure 16 (now Figure 15) was modified to a top 10, Figure 19 (now 18) was also modified, and finally, Figures 17 (now 16) and 18 (now 17) have been improved in resolution for better visualization, as restricting or grouping the data could be misinterpreted by aggregating publications from countries in a single region.

***Reviewer Comment:*** Correcting Potential errors: Looking up the single article included from 1998 (Zara Yahni, 1998), the article cites 43 papers, however, Table 1 mentions that 34 total studies were included in the RSL (which I assume is a typo for SLR). Thanks to your comments, we have reanalyzed the data in our manuscript and corrected Table 1, both in the data you observed and in the final value of the column, agreeing that it was a typographical error.

 Finally, we express our sincere thanks for the rigor and thoroughness of your review, which have been essential to make significant improvements in our work. Your highly constructive observations have been decisive in the review process, and we acknowledge with great appreciation the dedication and expertise embodied in your comments, which have greatly enriched the quality of our work.

# Reviewer report: Reviewer response for version 1

**DOI:** 10.5256/f1000research.171596.r352101

**Published:** 2025-01-10

**Authors:** Churchill Akena

**Affiliations:** 1 Pharmacy, Makerere University School of Health Sciences (Ringgold ID: 373383), Kampala, Central Region, Uganda

This paper discusses the implication of scientific knowledge on the performance efficiency of health systems worldwide. The authors argue that besides the cost and availability of health commodities, other factors related to the production of scientific literature impact health systems' ability to be efficient and sustainable in the long run. With this, the authors approached the argument with a key imperative objective: “to explore the panorama of the world scientific production of Open Access articles related to the prescription and dispensing of medicines and their relationship with the pharmaceutical expenditure faced by health systems”.

 The authors used scientometric mapping, a “research method based on statistical and visualization methods to represent critical points and trends in a specific field” to achieve this. The authors used open-access articles without any limitations to the publication date as long they were systematic literature reviews on the “analysis of prescription, dispensing, incentives and pharmaceutical expenditure of medicines in a health care setting”. To answer their research questions, the authors used the five main stages of scientometric mapping, these include: recovery or selection of sources, migration or extraction of data, analysis, visualization, and interpretation. The authors focused on key aspects: Databases and publications, Authors, Citations, Journals and Publications, Institutions and Countries, Funders and Sponsors, Co-occurrence of terms, and Research trends.

 In their findings, 103 articles were considered for final analysis. Under databases and publications, the first publication to meet the topic of this article was published in 1998 and is the only article from that year. There was a hiatus of research on the topic until 2006 when another article got published. There was then a steady rise in articles from 2010 until 2022. This article found that although systematic literature reviews (SLRs) had an initial hit of over 720,000 articles, less than 1% of these are considered in the final analysis. Concerning publications, the majority of these SLRs tend to be concentrated in the top research databases like PUBMED, Cochrane, and Epistemonikos.

 Under authors, this study found that, out of the 517 authors considered, the majority of the authors (94%) had low productivity index (PN = 0), a few (6%) with medium productivity (PN = >0<1) and none (0%) with high productivity (PN = >1) according to the Lotka’s law of productivity of authors. Regarding authors' collaboration, this article reports that the majority (67%) of the SLRs had between three to six authors, and one article had 32 authors overall.

 This article reported limitations in quantifying the number of citations per author. The authors, therefore, reported the articles and country of publications contributing the most citations. They found that 80% of the citations are due to 8 articles belonging to three single authors. Conversely, the United Kingdom (UK) had the highest number of citations (2600) by country from five publications, followed by the United States (1800) from three publications.

 Under Journals and publications, this study found that articles were published in journals roughly following Bradford’s law which states “that the production of articles in journals presents an uneven distribution, where a high number of articles are concentrated in a small number of journals and, conversely, a small number of articles are distributed in a large number of journals”. As their findings suggest, only journals had 33.98% of the articles published, which are Cochrane, PLoS One, and BMJ Open journals. A large proportion of these publications were concentrated in the UK, with at least 50% from the Western region of continental Europe which the UK is a part of. In addition, the majority of these journals (70%) were classified as high-impact journals based on their assigned impact factor.

 With regards to institutions and countries, this study found that the largest proportion (80%) of the authors were university affiliates, with government entities closely following (8%). As for countries, most of the authors (>50%) were from four countries; the UK, the US, Canada, and Australia. In addition, the authors of this study further visualized the relationship between the country of the primary studies and the country of SLR for these studies. They found that the majority of primary studies cover a wider geographical area irrespective of the country of publication of the SLR.  Furthermore, lead authors from the US tend to use primary studies conducted in the US for their SLR. This was not the case for authors from Qatar who tended to use primary studies from a wider variety of countries in their SLR.

 Under funders and sponsors, this study found that 30% of the articles reviewed did not provide information about their sponsors. For those that did, government entities were the highest proportion (25%) of the sponsors, followed by university institutions (8%). This article further characterized the country of affiliation of the sponsors and that of the authors. They found in the US and UK, authors affiliated with these countries have the same affiliation as their sponsors and funders.

 The authors of this article found that the most frequently mentioned terms in their review articles were Patients, Outcomes, Evidence, Prescribing, and COVID.

 When reviewing research trends, it was found that 50% of the studies did not mention a particle drug category whereas 14% mentioned antibiotics, 8% opioids, and 2% mentioned generic and oncology medicines.

 In their discussion of the findings, the authors presented key arguments to enhance understanding of what their results imply.

 For the observed trends in the number of articles published per year, this study argues that the recent rise in the trend is due to more recent publications in public health, more innovation, and easier access to publications due to wider internet coverage.

 This study rationalizes that less than 1% of the initial hits of articles are considered in systematic reviews due to their rigorous nature, ensuring a more reliable result.

 The authors contest that low specialization is responsible for the low productivity observed according to Lotka’s productivity index for authors. They advise more collaboration among authors to improve this observation.

 The concentration of published literature in journals with high impact factors could be due to an obsession for recognition by some universities and researchers wanting high research indicators. The authors of this article emphasize caution when considering impact factors for the judgment of research quality.

 In their judgment of why most publications are in European and American journals, the authors suggest a relationship with the need to publish in high-impact journals that are coincidentally located in these countries. Furthermore, the authors suggest the need to move away from “scientific colonialism” and encourage more publication in other countries, especially if the primary researches are carried out in those countries.

 With regards to funding, the authors of this study rationalize that high-income countries are likely to have more income to fund studies conducted by authors affiliated with them than low-income countries. In addition, they add that the nondisclosure of funders in some studies is to avoid being judged about the possibility of their study findings being influenced by their funders, especially if the funders are private organizations.

 When discussing the need for the co-occurrence of terms, the authors note that these terms are related to topics on knowledge and analysis of health expenditures which are important for the financial sustainability of healthcare systems.

 The authors argue that the predominant mention of antibiotics is related to conservation efforts under the umbrella of antimicrobial stewardship programs enacted worldwide to curb the rise in antimicrobial resistance. Opioids are mentioned predominantly due to the deemed opioid epidemic in the US warranting regular safety updates from WHO.

 In conclusion, this study recognizes the importance of scientometric mapping in gathering evidence from diverse research databases globally and shaping policies that support sustainable health systems.

 The authors acknowledge the limitations of relying solely on open-access articles in their systematic literature review. This approach restricts the inclusion of articles that are not freely accessible and may overlook key terms not mentioned in other studies. Furthermore, the authors note that focusing on annual publications can lead to an underestimation of relevant findings.

**Reviewer’s feedback**

**Introduction: **In paragraph three opening sentence, the authors start by saying, “Instead of “looking for” conventional market analysis factors such as frequency and price, or those related to a holistic and etiological approach to prescribing”. This suggests that the factors discussed in this review are in contrast to those related to pricing and other causal approaches to prescribing and dispensing, which is not entirely accurate. I recommend that the authors rephrase to say, “In addition to… we delve further into…” because the other factors mentioned later, such as unfair competition, complement rather than contradict the previously discussed factors.

**Methods: **The research design is appropriate for this study and the method is succinctly described to allow for the study replication. Although the study provides a clear rationale for considering only open-access articles as well as using Systematic review literature in this study, it is important to note that bias can still have a profound effect on the outcome. There is still selection bias as well as bias carried forward in the Systematic literature reviews from their primary studies which should be considered in the contexts of these findings (1). Perhaps a statement about this under the study limitations can assuage readers’ concern about this possibility as one can wonder if the results can have an entirely different picture if primary studies were included, for example.

**Results: **The results are informative and backed up by the study objectives and methodology.

**Discussions: **The discussions effectively address the study's findings. The authors highlight the key results of the research. However, it is essential to consider how the number of citations can impact the perceived quality of a study. The authors mentioned that “citations do not necessarily correspond to the academic recognition of publications”. While this statement holds some truth, several factors can help someone assess the quality of a study, which in turn may lead to a higher number of citations compared to other studies. Key factors include the length and wording of the title, the choice of keywords, and the content and length of the abstract, among others. (2). It would be important for the authors to recognize this in their argument and provide a sense of direction for the readers.

**Conclusions: **The study concludes based on its findings and discussions. The conclusions do not overstate the significance of the findings underlined.

 Overall, I believe this study takes a definitive scientific approach to addressing the concerns outlined in the study’s objectives and questions. My concerns are minor and do not affect the scientific foundation of this research. I sincerely applaud the authors for synthesizing a vast amount of data and bringing valuable evidence to the forefront of the scientific community.

 1. Drucker et al., 2016 (Ref 2) 

 2. Kousha K et al., 2024 (Ref 1)

Is the work clearly and accurately presented and does it cite the current literature?

Yes

If applicable, is the statistical analysis and its interpretation appropriate?

Yes

Are all the source data underlying the results available to ensure full reproducibility?

Yes

Is the study design appropriate and is the work technically sound?

Yes

Are the conclusions drawn adequately supported by the results?

Yes

Are sufficient details of methods and analysis provided to allow replication by others?

Yes

Reviewer Expertise:

Pharmaceutical science, public health

I confirm that I have read this submission and believe that I have an appropriate level of expertise to confirm that it is of an acceptable scientific standard.

## References

[1] : Factors associating with or predicting more cited or higher quality journal articles: An Annual Review of Information Science and Technology (ARIST) paper. *Journal of the Association for Information Science and Technology* .2024;75(3) : 10.1002/asi.24810 215-244 10.1002/asi.24810

[2] : Research Techniques Made Simple: Assessing Risk of Bias in Systematic Reviews. *J Invest Dermatol* .2016;136(11) : 10.1016/j.jid.2016.08.021 e109-e114 10.1016/j.jid.2016.08.021 27772550

# Response

**Published:** 2025-01-28

**Authors:** Luis Hernando Tocaruncho Ariza

**Affiliations:** University of Bogota Jorge Tadeo Lozano Faculty of Natural Sciences and Engineering, Bogotá, Bogota, Colombia

Title: “Incentives in prescribing, dispensing and pharmaceutical spending: A scientometric mapping.

 Tocaruncho-Ariza L. H, Riascos-Ochoa J, Jimenez-Barbosa W. G

 Dear Churchill Akena,

 We sincerely appreciate the careful review and constructive suggestions you have provided, which have been extremely helpful in identifying areas of improvement and enriching our work. Your detailed feedback has been crucial to optimize both the presentation and content of our article, and we deeply appreciate your thorough analysis and recognition of the contribution of the manuscript to the scientific community.

 In the following, we address all the raised issues and also summarize the changes made in the manuscript.

  

1. ***Introduction:*** *In paragraph three opening sentence, the authors start by saying, “Instead of “looking for” conventional market analysis factors such as frequency and price, or those related to a holistic and etiological approach to prescribing”. This suggests that the factors discussed in this review are in contrast to those related to pricing and other causal approaches to prescribing and dispensing, which is not entirely accurate. I recommend that the authors rephrase to say, “In addition to… we delve further into…” because the other factors mentioned later, such as unfair competition, complement rather than contradict the previously discussed factors.*

We fully agree with your feedback and have made the adjustment suggested in the Introduction section. We hope we have met your expectations.

  

1. *Methods: The research design is appropriate for this study and the method is* *succinctly described to allow for the study replication. Although the study provides a clear rationale for considering only open-access articles as well as using Systematic review literature in this study, it is important to note that bias can still have a profound effect on the outcome. There is still selection bias as well as bias carried forward in the Systematic literature reviews from their primary studies which should be considered in the contexts of these findings (1). Perhaps a statement about this under the study limitations can assuage readers’ concern about this possibility as one can wonder if the results can have an entirely different picture if primary studies were included, for example.*

We deeply appreciate your critical comments, which were essential to present the limitations of our manuscript, so we have included in the limitations section those related to selection bias and others related to literature review, highlighting the caution in generalizing the findings.

  

1. ***Discussions:*** *The discussions effectively address the study's findings. The authors highlight the key results of the research. However, it is essential to consider how the number of citations can impact the perceived quality of a study. The authors mentioned that “citations do not necessarily correspond to the academic recognition of publications”. While this statement holds some truth, several factors can help someone assess the quality of a study, which in turn may lead to a higher number of citations compared to other studies. Key factors include the length and wording of the title, the choice of keywords, and the content and length of the abstract, among others. (2). It would be important for the authors to recognize this in their argument and provide a sense of direction for the readers.*

We greatly appreciate the valuable suggestions provided in your review, and in response to your recommendation, we have included in the Discussion section some factors that affect the number of citations of a publication, thus broadening the discussion of citations for the reader to consider.

 Best regards,

 The authors,

 Luis Hernando Tocaruncho Ariza

 Javier Riascos Ochoa

 Wilson Giovanni Jiménez Barbosa
